# Tissue Transglutaminase (TG2) as a Candidate Biomarker and Potential Therapeutic Target in Endometriosis: Functional Evidence from shRNA-Mediated Downregulation of TG2 in Endometrial Mesenchymal Stem Cells

**DOI:** 10.3390/ph19101520

**Published:** 2026-09-25

**Authors:** İnci Kurt-Celep, Melike Batukan, Fikrettin Şahin, Dilek Telci

**Affiliations:** 1 Department of Genetics and Bioengineering, Yeditepe University, 26 August Campus, Kayisdagi, Atasehir, Istanbul 34755, Türkiye; inci.celep@okan.edu.tr (İ.K.-C.); melikebatukan@yahoo.com (M.B.); fsahin@yeditepe.edu.tr (F.Ş.); 2 Department of Biotechnology, Faculty of Pharmacy, Istanbul Okan University, Tuzla, Istanbul 34959, Türkiye

**Keywords:** endometriosis, endometrial mesenchymal stem cells (eMSCs), tissue transglutaminase (TG2), TG2–ITGβ-1/SDC-4 associations, matrix metalloproteinases, cell cycle progression, shRNA-mediated knockdown

## Abstract

**Background/Objectives**: Endometriosis involves the presence of endometrial tissue outside the uterus. Since the migration of endometrial cells is pivotal to lesion development, tissue transglutaminase (TG2)—known for its roles in cell adhesion, migration, and stemness—may play a significant role. This study aimed to investigate the involvement of TG2 in the migratory and invasive characteristics of endometrial mesenchymal stem cells (eMSCs) derived from healthy individuals and endometriosis patients. **Methods**: eMSCs were isolated from healthy controls (heMSCs) and endometriosis patients (peMSCs). TG2 protein expression and enzyme activity were evaluated. Co-immunoprecipitation (Co-IP) experiments were performed to assess TG2 associations with ITGβ-1 and SDC-4 in membrane fractions. Additionally, shRNA technology was utilized to silence TG2 in peMSCs to analyze its effects on cell proliferation, migration, invasion, and matrix metalloproteinase (MMP-2 and -9) activities. **Results**: peMSCs exhibited higher TG2 protein expression but lower enzyme activity compared to heMSCs. Co-IP analysis of membrane fractions revealed enhanced association of TG2 with ITGβ-1 and SDC-4 in peMSCs, thereby promoting enhanced cell survival, migration, and invasive properties. Silencing TG2 in peMSCs via shRNA eliminated these enhanced proliferation, migration, and invasion capabilities, reverting them to a phenotype resembling heMSCs. Furthermore, elevated MMP-2 and -9 activities returned to basal levels following TG2 knockdown. **Conclusions**: These findings suggest that TG2 acts as a distinctive cell adhesion molecule directly involved in endometriosis pathogenesis. Collectively, these findings identify TG2 as a disease-associated molecular marker warranting further evaluation for its diagnostic potential and suggest that targeting its cell-adhesion functions may represent a novel therapeutic strategy for endometriosis.

## 1. Introduction

Endometriosis is a disease characterized by the presence of endometrial-like tissue outside the uterine cavity in women of reproductive age [1]. Endometriosis takes its name from the endometrium, and although the disease was first described in 1860 [2] its etiology and pathogenesis remain unclear [3]. In addition to its gynecological manifestations, endometriosis has been associated with several comorbid conditions, including migraine [4]. There are various classifications and theories regarding the pathophysiology of endometriosis [1,5]. The most widely accepted theory suggests that the disease develops through the dissemination of endometrial tissue into the peritoneal cavity through retrograde menstruation, as proposed by Sampson in the 1920s [6,7]. The refluxed fragments of endometrial tissue cause the disease by attaching to the peritoneal surface, where they establish ectopic lesions through invasion [7]. According to Sampson, there were still viable cells in menstrual blood meant to be expelled with each monthly cycle. These cells had the ability to migrate to ectopic sites, adhere to surrounding tissues, and proliferate. However, the molecular mechanisms underlying these processes could not be explained at that time [5,6]. The endometrium comprises various cell populations, including fibroblasts, endothelial cells, smooth muscle cells, and mesenchymal stem cells [8,9]. In women developing endometriosis, it is hypothesized that the endometrial stem cells (eMSCs), which would normally be shed during menstruation, are transported into the peritoneal cavity together with their niche cells through retrograde menstruation, supporting Sampson’s theory [10,11]. However, the molecular mechanism underlying the migratory and adhesive properties of eMSCs remain poorly understood. The enhanced adhesive and migratory properties of eMSCs suggest that tissue transglutaminase (TG2) may regulate these processes [12,13,14].

TG2, a member of the transglutaminase family, is a multifunctional protein that possesses both Ca^2+^-dependent transamidase activity and GTP-binding/hydrolyzing activity [15,16]. Through its transamidase activity, TG2 catalyzes protein cross-linking by forming ε-(γ-glutamyl) lysine isopeptide bonds, thereby contributing to extracellular matrix (ECM) stabilization, wound healing, angiogenesis, and bone repair [16,17]. In addition to its enzymatic functions, TG2 has important non-enzymatic roles in cell adhesion and migration by interacting with integrins (containing β-1, β-3, and β-5), syndecan-4, and fibronectin to form cell surface adhesion complexes that promote cell attachment to the ECM [14,18,19]. Recent studies have demonstrated that extracellular TG2 functions as a co-receptor for integrins and syndecan-4 independently of its transamidase activity, thereby protecting cells from anoikis while promoting cell adhesion, migration, and survival [20,21]. Consistent with these observations, TG2 overexpression has been associated with enhanced metastatic potential and drug resistance in several malignancies, including melanoma and breast, lung, and pancreatic cancers [22,23]. In addition to its role in cell adhesion, TG2 regulates ECM remodeling by promoting NF-κB-dependent TGF-β1 activation, resulting in increased ECM protein synthesis and deposition [20,24].

Menstrual shedding of the endometrium is mediated by ECM degradation through matrix metalloproteinases (MMPs) together with apoptosis of endometrial cells [25,26,27]. Likewise, the establishment of endometriotic lesions requires the adhesion of endometrial fragments to the peritoneal surface, followed by remodeling of the peritoneal mesothelium, processes that depend on MMP activation [11]. MMPs facilitate both the breakdown and regeneration of the endometrium under estrogen stimulation, and increased MMP expression has been associated with the enhanced invasive potential of ectopic endometrial tissue, thereby contributing to the development of endometriosis [11,27]. Recent studies have demonstrated that TG2 regulates ECM remodeling through TGF-β1 signaling. Furthermore, inhibition of its transamidase activity alters MMP expression profiles and is associated with enhanced cell migration [24,28]. Considering the increased MMP expression and activity observed in endometriosis, together with the regulatory role of TG2 in MMP activation, TG2 may represent a key mediator in the development of endometriosis.

Although TG2 has been extensively studied in cancer progression and ECM remodeling, its role as a cell adhesion molecule in the pathogenesis of endometriosis remains largely unexplored. Therefore, in the present study, we investigated the role of TG2 in the development of endometriosis using eMSCs isolated from healthy individuals (heMSCs) and patients with endometriosis (peMSCs). Surface marker profiles were characterized by flow cytometry [29], and TG2 expression and transamidase activity were evaluated in both cell populations. In addition, the interactions of TG2 with integrin β-1 and syndecan-4, MMP-2 and MMP-9 activity, and the effects of TG2 silencing on cell proliferation, migration, and invasion were investigated to elucidate the contribution of TG2 to the pathological phenotype of endometriosis-derived eMSCs.

## 2. Results

### 2.1. Phenotypic Characterization of Healthy and Endometriosis-Derived eMSCs

eMSCs isolated from healthy individuals (heMSCs, *n* = 5) and patients with endometriosis (peMSCs, *n* = 5) were characterized by flow cytometry using mesenchymal stem cell and hematopoietic surface markers (Figure 1). Representative histograms from all independent donors are shown in Supplementary Figures S1 and S2.

Both heMSCs and peMSCs displayed the characteristic immunophenotype of mesenchymal stem cells, exhibiting high expression of the canonical MSC markers CD44, CD29, CD73, CD90, CD105, and integrin β-1 (ITGβ-1), while hematopoietic/endothelial markers (CD14, CD31, CD34, and CD45) remained below 3% in both groups (Figure 1 and Table 1).

In contrast, the perivascular/progenitor-associated markers CD146, W5C5, and PDGFR were significantly enriched in peMSCs compared with heMSCs. CD146 expression increased from 42.7% ± 3.1 in heMSCs to 75.5% ± 2.5 in peMSCs (*p* ≤ 0.0001), W5C5 from 44.6% ± 2.4 to 74.0% ± 6.3 (**** *p* ≤ 0.0001), and PDGFR from 43.4% ± 4.1 to 74.0% ± 2.3 (*p* ≤ 0.0001) (Table 1). No significant differences were observed in the expression of CD44, CD29, ITGβ-1, CD73, CD90, CD105, or the hematopoietic/endothelial markers between the two groups. Together, these findings demonstrate that although both cell populations retained their mesenchymal stem cell identity, peMSCs exhibited a distinct perivascular/progenitor-associated phenotype compared with heMSCs.

### 2.2. TG2 Expression and Transamidase Activity in Healthy and Endometriosis-Derived eMSCs

To determine whether TG2 expression and activity are altered in endometriosis, TG2 mRNA expression, protein levels, and transamidase activity were evaluated comparatively in heMSCs and peMSCs (Figure 2). RT-qPCR analysis normalized to 18S rRNA revealed a significant upregulation of TG2 mRNA expression in peMSCs compared with heMSCs (Figure 2A). TG2 transcript levels were consistently elevated across the five independently derived peMSC samples showing an approximately 3- to 5-fold increase relative to healthy donor-derived eMSCs. When the data from all donor-derived eMSCs were analyzed collectively, TG2 mRNA expression remained significantly higher in peMSCs than in heMSCs (*p* ≤ 0.001), demonstrating a consistent transcriptional upregulation of TG2 in endometriosis-derived eMSCs.

Consistent with the RT-qPCR findings, Western blot analysis demonstrated higher TG2 protein levels in peMSCs than in the heMSCs across the five independent donor-derived samples in each group (G1–G5) (Figure 2B). Representative immunoblots showed a marked increase in the intensity of the TG2 protein bands in peMSCs, while β-actin was used as a loading control. Densitometric analysis normalized to β-actin confirmed that TG2 protein expression was consistently elevated in peMSCs, showing an approximately 4- to 5-fold increase relative to healthy donor-derived eMSCs (*p* ≤ 0.0001). These findings demonstrate that the transcriptional upregulation of TG2 in endometriosis-derived eMSCs is accompanied by a corresponding increase in TG2 protein expression.

To determine whether the increased TG2 expression observed in peMSCs was accompanied by enhanced enzymatic activity, TG2 transamidase activity was measured using the BTC incorporation assay under Ca^2+^-dependent conditions (Figure 2C). In contrast to the mRNA and protein expression data, TG2 transamidase activity was consistently reduced in peMSCs compared with the heMSCs across the five independent donor-derived samples in each group (G1–G5). Whereas heMSCs exhibited robust TG2 transamidase activity, peMSCs displayed markedly diminished activity, approaching background levels. Quantitative analysis confirmed a significant reduction in TG2 transamidase activity in peMSCs relative to heMSCs (*p* ≤ 0.0001), despite the marked increase in TG2 mRNA and protein expression.

Collectively, these findings demonstrate that although TG2 expression is markedly increased in endometriosis-derived eMSCs, its transamidase activity is significantly diminished, indicating that TG2 predominantly exists in a non-enzymatically active state.

### 2.3. Enhanced MMP-2 and MMP-9 Expression and Activity in Endometriosis-Derived eMSCs

To investigate whether the increased invasive phenotype of peMSCs is associated with altered matrix metalloproteinase expression, MMP-2 and MMP-9 mRNA expression and enzymatic activity were evaluated in heMSCs and peMSCs (Figure 3). RT-qPCR analysis revealed significantly higher MMP-2 and MMP-9 mRNA expression in peMSCs than in the heMSCs (G1–G5) (Figure 3A). Across the five independently derived peMSC samples, both MMP-2 and MMP-9 transcript levels were consistently elevated in peMSCs relative to the heMSC group. Similarly, MMP-2 transcript levels were consistently increased by approximately 2.5- to 4-fold in peMSCs relative to the heMSCs group. Similarly, MMP-9 mRNA expression was consistently elevated in peMSCs, with fold increases ranging from approximately 4- to 5-fold compared with the healthy controls. When the data from all donor-derived eMSCs were analyzed collectively, both MMP-2 and MMP-9 mRNA expression levels remained significantly higher in peMSCs than in heMSCs (*p* ≤ 0.001), demonstrating a consistent transcriptional upregulation of both metalloproteinases in endometriosis-derived eMSCs.

To determine whether the increased MMP-2 and MMP-9 mRNA expression was accompanied by enhanced enzymatic activity, zymographic analysis was performed using conditioned media collected from heMSCs and peMSCs (Figure 3B). Distinct lytic bands corresponding to MMP-2 and MMP-9 were detected in all samples. Compared with heMSCs, peMSCs consistently exhibited stronger lytic band intensities across the independently derived donor samples, indicating increased MMP-2 and MMP-9 activity. Densitometric analysis of the zymograms confirmed a significant increase in the enzymatic activities of both MMP-2 and MMP-9 in peMSCs relative to heMSCs, with an overall increase of approximately 3- to 5-fold (**** *p* ≤ 0.0001). Together, these findings demonstrate that the transcriptional upregulation of MMP-2 and MMP-9 in endometriosis-derived eMSCs is accompanied by a corresponding increase in their enzymatic activity.

### 2.4. TG2 Interaction with Integrin β1 and Syndecan-4 in Endometriosis-Derived eMSCs

To investigate the interaction of TG2 with its known cell surface binding partners, integrin β-1 (ITGβ-1) and syndecan-4 (SDC-4), co-immunoprecipitation analysis was performed using heMSCs and peMSCs (Figure 4). ITGβ-1 and SDC-4 were specifically selected because of their established functional cooperation with TG2 and fibronectin in the regulation of cell-matrix adhesion and migration [14].

Comparable levels of ITGβ-1 were detected in the input fractions of both heMSCs and peMSCs (Figure 4A). In contrast, the amount of TG2 co-immunoprecipitated with ITGβ-1 was substantially increased in peMSCs. Quantitative analysis demonstrated that the TG2/ITGβ-1 interaction ratio increased from 0.006 ± 0.005 in heMSCs to 5.6 ± 1.7 in peMSCs (*p* ≤ 0.0001). This increase was consistently observed across the peMSC samples (G1–G5), indicating enhanced interaction between TG2 and ITGβ-1 in endometriosis-derived eMSCs.

A similar pattern was observed for syndecan-4 (Figure 4B). Although SDC-4 input levels were comparable between the two groups, substantially higher amounts of TG2 co-immunoprecipitated with SDC-4 in peMSCs than in heMSCs. Quantitative analysis showed that the TG2/SDC-4 interaction ratio increased from 0.014 ± 0.001 in heMSCs to 1.2 ± 0.1 in peMSCs (*p* ≤ 0.0001). Together, these findings demonstrate enhanced membrane-associated association of TG2 with ITGβ-1 and SDC-4 in peMSCs.

### 2.5. Cell Cycle Progression and Enhanced Proliferation in Endometriosis-Derived eMSCs

To determine whether the altered molecular profile of peMSCs was associated with changes in cell proliferation and cell cycle progression, cell growth kinetics and the expression of cell cycle regulatory proteins were evaluated (Figure 5). Analysis of cell proliferation demonstrated that peMSCs exhibited a significantly higher proliferation rate than heMSCs throughout the 96 h observation period (Figure 5A). Both cell populations were initially seeded at a density of 2000 cells/well. After 24 h, heMSCs remained close to the initial seeding density (2038 ± 61 cells), whereas peMSCs had already increased to 2372 ± 78 cells (** *p* ≤ 0.01), indicating an earlier onset of proliferation. This proliferative advantage became progressively more pronounced over time, with peMSCs consistently exhibiting significantly higher cell numbers than heMSCs at 48, 72, and 96 h (**** *p* ≤ 0.0001). By the end of the experiment, peMSCs had expanded approximately 4.2-fold relative to the initial seeding density, whereas heMSCs exhibited an approximately 3.3-fold increase, demonstrating a sustained proliferative advantage in endometriosis-derived eMSCs.

To investigate the molecular basis underlying the increased proliferative capacity of peMSCs, the expression of key cell cycle regulatory proteins was analyzed by Western blotting (Figure 5B). Western blot images obtained from all five groups (G1–G5) demonstrated a consistent increase in the expression of proteins that promote G1/S cell cycle progression, including Cyclin E1, Cyclin D3, CDK2, and CDK4, in peMSCs compared with heMSCs, whereas the cell cycle inhibitor p27 was consistently reduced. Densitometric analysis confirmed a significant upregulation of Cyclin E1 (3.6 ± 0.3 vs. 1.2 ± 0.3), CDK2 (2.4 ± 0.4 vs. 1.0 ± 0.3), Cyclin D3 (2.4 ± 0.5 vs. 0.8 ± 0.4), and CDK4 (2.0 ± 0.4 vs. 0.5 ± 0.3) in peMSCs relative to heMSCs (**** *p* ≤ 0.0001). In contrast, p27 expression was significantly decreased in peMSCs (0.4 ± 0.1) compared with heMSCs (1.1 ± 0.4; ** *p* ≤ 0.01). Together, these findings demonstrate that the enhanced proliferative phenotype of endometriosis-derived eMSCs is accompanied by coordinated upregulation of proteins involved in G1/S cell cycle progression and downregulation of the cell cycle inhibitor p27.

### 2.6. shRNA-Mediated Silencing of TG2 in Endometriosis-Derived eMSCs

To investigate the functional contribution of TG2 to the endometriosis-associated phenotype, TG2 was selectively silenced in peMSCs using shRNA, and TG2 protein expression was evaluated by Western blot analysis (Figure 6).

Western blot analysis demonstrated that TG2 protein expression was markedly higher in peMSCs than in heMSCs (Figure 6A), consistent with the findings presented in Figure 2B. Treatment with the scramble (SCR) control did not alter TG2 protein expression in either cell population, as TG2 levels remained low in heMSCs and high in peMSCs, confirming that the scramble construct had no detectable effect on TG2 expression. In contrast, shRNA-mediated silencing markedly reduced TG2 protein expression in peMSCs to levels comparable with those observed in heMSCs. Quantitative densitometric analysis of TG2 protein expression normalized to β-actin showed that TG2 protein levels were low in heMSCs (0.5 ± 0.3) and were not significantly altered following SCR treatment (0.3 ± 0.2, ns) (Figure 6B). Likewise, the elevated TG2 expression observed in peMSCs remained unchanged after SCR treatment (2.6 ± 0.9), confirming that the SCR construct did not affect TG2 protein expression. In contrast, shRNA-mediated silencing reduced TG2 protein levels in peMSCs to 0.3 ± 0.3, which was not significantly different from either untransduced or SCR-transduced heMSCs (ns). This expression pattern was consistently observed across the five independent donor-derived samples in each group (G1–G5), demonstrating reproducible TG2 silencing across biological replicates. The overall comparison of the four experimental groups (Figure 6C) confirmed that TG2 expression remained significantly elevated in peMSCs following SCR treatment (**** *p* ≤ 0.0001), whereas shRNA-mediated silencing restored TG2 protein levels to those observed in healthy controls (ns). TG2 mRNA expression was significantly increased in peMSCs + SCR and markedly reduced following TG2 shRNA-mediated knockdown. Individual TG2 mRNA expression values for the five independent donor-derived samples in each group (G1–G5), together with the corresponding group means, are presented in Supplementary Figure S3.

### 2.7. TG2 Silencing Modulates Endometriosis-Associated Surface Marker Expression in eMSCs

To determine whether TG2 contributes to the altered immunophenotype of endometriosis-derived eMSCs, surface marker expression was analyzed by flow cytometry following shRNA-mediated TG2 silencing (Figure 7). Representative histograms are shown in Figure 7, whereas quantitative data are summarized in Table 2. Individual flow cytometry profiles from the five independent donor-derived samples in each group are provided in Supplementary Figures S4 and S5.

As shown in Figure 7 and summarized in Table 2, SCR treatment did not alter the immunophenotype of either heMSCs or peMSCs. No significant differences were observed between untreated and SCR-transduced heMSCs in the expression of CD146, W5C5, PDGFR, the canonical MSC markers (CD44, CD29, ITGβ-1, CD73, CD90, and CD105), or the hematopoietic markers (CD14, CD31, CD34, and CD45) (Figure 7A). Likewise, the elevated expression of CD146, W5C5, and PDGFR observed in peMSCs remained unchanged following SCR treatment, while the expression of the canonical MSC and hematopoietic markers was also unaffected (Figure 7B). These findings confirm that the SCR construct does not alter the immunophenotypic characteristics of either healthy or endometriosis-derived eMSCs.

In contrast, TG2 silencing markedly altered the immunophenotype of peMSCs (Figure 7B). CD146 expression decreased from 71.4 ± 5.1% to 35.5 ± 5.4%, W5C5 from 76.6 ± 2.5% to 42.1 ± 3.4%, and PDGFR from 78.5 ± 0.5% to 34.8 ± 4.0% following TG2 shRNA treatment (**** *p* ≤ 0.0001) (Table 2). In contrast, the expression of canonical MSC markers (CD44, CD29, ITGβ-1, CD73, CD90, and CD105) and hematopoietic markers (CD14, CD31, CD34, and CD45) remained unchanged following TG2 silencing. Together, these findings demonstrate that TG2 regulates the expression of the endometriosis-associated surface markers CD146, W5C5, and PDGFR without altering the fundamental mesenchymal stem cell phenotype.

### 2.8. TG2 Silencing Reduces Cell Migration in Endometriosis-Derived eMSCs

To determine whether TG2 contributes to the enhanced migratory phenotype of endometriosis-derived eMSCs, cell migration was evaluated using a wound healing assay (Figure 8). Representative wound healing images obtained from all five independent donor-derived samples in each group at 0, 6, and 12 h are presented in Supplementary Figure S6. All experimental groups exhibited comparable wound widths at baseline (0 h) (Figure 8A). During the subsequent incubation period, heMSCs and heMSCs + SCR displayed limited wound closure, whereas peMSCs and peMSCs + SCR migrated rapidly into the wound area, with substantial closure evident after 6 h and nearly complete closure after 12 h. In contrast, TG2 silencing markedly reduced the migratory capacity of peMSCs, resulting in a wound closure pattern comparable to that observed in both untreated and SCR-transduced heMSCs.

Quantitative analysis of wound closure is presented in Figure 8B. After 6 h, both peMSCs (32.7 ± 5.3%) and peMSCs + SCR (32.7 ± 4.7%) exhibited significantly greater wound closure than heMSCs (4.0 ± 1.1%) and heMSCs + SCR (4.1 ± 0.9%) (**** *p* ≤ 0.0001). TG2 silencing almost completely abolished this enhanced migratory phenotype, reducing wound closure to 5.3 ± 1.2%, a level comparable to that of healthy controls. By 12 h, wound closure was nearly complete in peMSCs (100%) and peMSCs + SCR (98.9 ± 1.5%), whereas heMSCs (6.3 ± 1.7%), heMSCs + SCR (6.8 ± 1.8%), and peMSCs + shRNA (5.8 ± 1.3%) exhibited only minimal migration. Wound closure in TG2-silenced peMSCs did not differ significantly from either untreated or SCR-transduced heMSCs (ns). Together, these findings demonstrate that TG2 contributes to the enhanced migratory capacity of endometriosis-derived eMSCs.

### 2.9. TG2 Downregulation Attenuates MMP-2 and MMP-9 Expression and Activity in Endometriosis-Derived eMSCs

To determine whether TG2 contributes to the elevated MMP-2 and MMP-9 expression and activity observed in endometriosis-derived eMSCs, MMP-2 and MMP-9 mRNA expression and enzymatic activity were evaluated following TG2 silencing using RT-qPCR and gelatin zymography, respectively (Figure 9). Representative gelatin zymograms obtained from the five independent donor-derived samples in each group are presented in Supplementary Figure S7. Both MMP-2 and MMP-9 mRNA expression levels were markedly increased in peMSCs compared with heMSCs and remained unchanged following SCR treatment. MMP-2 expression increased approximately 2.5-fold in peMSCs and remained elevated in peMSCs + SCR (2.4-fold, ns), whereas MMP-9 expression increased approximately 3.2-fold and remained similarly elevated following SCR treatment (3.1-fold, ns). In contrast, TG2 downregulation markedly reduced the expression of both MMPs. MMP-2 expression decreased to 0.9-fold (approximately 2.9-fold lower than peMSCs), whereas MMP-9 expression decreased to 0.8-fold (approximately 3.3-fold lower than peMSCs), restoring both transcripts to levels comparable with those of heMSCs (**** *p* ≤ 0.0001).

Consistent with the transcriptional findings, gelatin zymography demonstrated significantly higher MMP-2 and MMP-9 activities in peMSCs than in heMSCs (Figure 9B). MMP-2 activity increased approximately 2.5-fold in peMSCs and remained elevated following SCR treatment (2.4-fold, ns), whereas MMP-9 activity increased approximately 1.7-fold and was similarly maintained in peMSCs + SCR (1.7-fold, ns). No significant differences were observed between parental and SCR-transduced heMSCs, confirming that the SCR construct had no effect on MMP activity. In contrast, TG2 downregulation markedly reduced the gelatinolytic activity of both MMPs. MMP-2 activity decreased approximately 2.9-fold, whereas MMP-9 activity decreased approximately 1.8-fold relative to peMSCs, restoring both enzymes to activity levels comparable with those observed in heMSCs (**** *p* ≤ 0.0001). Together, these findings demonstrate that TG2 contributes to the elevated MMP-2 and MMP-9 activity associated with the endometriotic phenotype.

### 2.10. TG2 Silencing Suppresses Proliferation and Restores Cell Cycle Progression in Endometriosis-Derived eMSCs

To determine whether TG2 downregulation affects the enhanced proliferative phenotype of endometriosis-derived eMSCs, cell proliferation was assessed by WST-1 assay at 24, 48, 72, and 96 h (Figure 10A). Individual Western blot images of cell cycle-related proteins and the corresponding cell cycle histograms obtained from the five independent donor-derived samples in each group are presented in Supplementary Figures S8 and S9, respectively. peMSCs exhibited significantly higher proliferation than heMSCs at all examined time points, with cell numbers consistently increasing by approximately 2- to 3-fold throughout the experimental period. This hyperproliferative phenotype remained unchanged following SCR transduction, confirming that the viral vector had no effect on cell growth. In contrast, TG2 downregulation markedly suppressed proliferation in peMSCs, restoring cell numbers to levels comparable with those observed in heMSCs throughout the experimental period (**** *p* ≤ 0.0001).

Consistent with these findings, Western blot analysis of the cell cycle regulatory proteins Cyclin E1, CDK-2, Cyclin D3, CDK-4, and p27 were performed across all experimental groups (Figure 10B). The elevated expression of Cyclin E1, CDK-2, Cyclin D3, and CDK-4 observed in peMSCs remained unchanged following SCR transduction but was markedly reduced following TG2 downregulation. Overall, the expression of these cell cycle-promoting proteins decreased by approximately 2- to 4-fold, returning to levels comparable with those observed in heMSCs (**** *p* ≤ 0.0001). No significant differences were detected between untreated and SCR-transduced peMSCs for any of the analyzed proteins (ns). In contrast, the reduced expression of the cyclin-dependent kinase inhibitor p27 in peMSCs was restored following TG2 downregulation, increasing by approximately 3-fold to levels comparable with those observed in heMSCs (**** *p* ≤ 0.0001).

To determine whether the changes observed in cell cycle regulatory proteins were reflected in functional cell cycle progression, cell cycle distribution was analyzed using the Cytell™ Cell Imaging System (Figure 10C). Compared with heMSCs, peMSCs exhibited a marked redistribution of cells from the G0/G1 phase to the S phase, with an approximately 2-fold reduction in the G0/G1 population accompanied by an approximately 4-fold increase in the S-phase fraction (**** *p* ≤ 0.0001). This altered cell cycle profile remained unchanged following SCR transduction. In contrast, TG2 downregulation restored cell cycle distribution by increasing the G0/G1 population by approximately 2-fold while reducing the S-phase fraction by approximately 2- to 3-fold, resulting in a cell cycle profile comparable with that observed in heMSCs (**** *p* ≤ 0.0001). Only minor variations were observed in the G2/M phase, with no consistent or biologically significant differences detected among the experimental groups. Together, these findings demonstrate that TG2 downregulation suppresses the hyperproliferative phenotype of endometriosis-derived eMSCs by restoring the expression of key cell cycle regulators and normalizing cell cycle progression.

### 2.11. TG2 Downregulation Reduces Clonogenic and Invasive Capacities in Endometriosis-Derived eMSCs

To determine whether TG2 downregulation influences the clonogenic and invasive capacities of endometriosis-derived eMSCs, colony-forming unit (CFU) and Transwell invasion assays were performed (Figure 11). The corresponding individual CFU and Transwell invasion images obtained from all the five independent donor-derived samples in each group are presented in Supplementary Figure S10. peMSCs exhibited a markedly enhanced clonogenic capacity compared with heMSCs, with colony formation increasing by approximately 50-fold, and this phenotype remained unchanged following SCR transduction (Figure 11A). In contrast, TG2 downregulation almost completely abolished the enhanced clonogenic phenotype, reducing CFU formation and restoring colony numbers to levels comparable with those observed in heMSCs (**** *p* ≤ 0.0001).

Consistent with the CFU findings, Transwell invasion analysis demonstrated that peMSCs exhibited a markedly enhanced invasive capacity compared with heMSCs (Figure 11B), with cell invasion increasing by approximately 9-fold and remaining unchanged following SCR transduction. In contrast, TG2 downregulation markedly suppressed cell invasion, reducing the number of invading cells and restoring invasive capacity to levels comparable with those observed in heMSCs (*p* ≤ 0.0001). Together, these findings demonstrate that TG2 downregulation effectively suppresses both the clonogenic and invasive phenotypes associated with endometriosis-derived eMSCs.

## 3. Discussion

Endometriosis is a chronic, multifactorial, and biologically complex gynecological disease characterized by the presence of endometrial-like tissue outside the uterine cavity [30]. Although Sampson’s theory proposes that viable endometrial fragments reach the peritoneal cavity through retrograde menstruation and subsequently implant at ectopic sites, this mechanism alone does not fully explain why only a subset of women develop persistent lesions despite the high prevalence of retrograde menstruation among women of reproductive age [31]. Thus, a critical question is not only how endometrial cells reach ectopic sites, but also which molecular mechanisms enable them to survive detachment, adhere to a foreign extracellular matrix, remodel their microenvironment, proliferate, migrate, invade, and ultimately establish persistent endometriotic lesions. Current models therefore recognize that endometriosis may involve multiple, potentially complementary mechanisms, including retrograde menstruation, stem/progenitor cell involvement, inflammatory microenvironmental cues, vascular or lymphatic dissemination, and lesion-specific tissue remodeling [11,32]. Moreover, endometriosis is not restricted to the pelvic cavity; extrapelvic manifestations, including thoracic involvement and, more rarely, central nervous system lesions, have been reported, suggesting that endometrial-derived cells may possess the capacity to disseminate, establish, and persist at distant ectopic sites [33]. In this context, the present study identifies TG2 as a functional regulator of the disease-associated phenotype of endometriosis-derived eMSCs and provides mechanistic insight into its contribution to cellular processes that may support ectopic lesion establishment and persistence.

The immunophenotypic profiles of both heMSCs and peMSCs were consistent with the established surface marker signature of endometrial mesenchymal stem/stromal cells reported previously [34,35]. The robust expression of standard MSC markers, together with negligible expression of hematopoietic and endothelial markers, confirms the identity of the isolated cell populations and supports the validity of the experimental model [35,36]. Beyond this shared MSC phenotype, peMSCs exhibited a distinct shift toward a perivascular/progenitor-associated profile, characterized by significantly increased expression of CD146, W5C5/SUSD2, and PDGFR. Co-expression of CD146 and PDGFR has previously been shown to identify a perivascular population of human endometrial stromal cells enriched for clonogenic and multipotent MSC-like properties [37], whereas W5C5/SUSD2 has been established as a single marker capable of enriching clonogenic endometrial mesenchymal stem/stromal cells [38]. These findings therefore suggest that endometriosis-derived eMSCs are enriched for a perivascular/progenitor-associated phenotype with increased clonogenic potential. Collectively, the present findings indicate that endometriosis is associated with a shift toward a progenitor/perivascular-associated phenotype that may favor the cellular plasticity required for ectopic implantation and lesion persistence. This phenotypic shift also provides a biological framework for investigating the functional role of TG2 in the disease-associated behavior of peMSCs.

One of the most significant findings of the present study is the dissociation between TG2 expression and its transamidase activity. Although peMSCs exhibited markedly increased TG2 mRNA and protein levels, Ca^2+^-dependent transamidase activity was significantly reduced compared with heMSCs. This apparent paradox suggests that increased TG2 expression in endometriosis-derived eMSCs is not accompanied by a corresponding increase in its classical Ca^2+^-dependent ε-(γ-glutamyl) lysine cross-linking activity [14,39,40,41]. These findings are consistent with the well-established conformational and functional regulation of TG2. Ca^2+^ binding favors an open, transamidase-active conformation, whereas guanine nucleotide binding stabilizes a closed conformation in which transamidase activity is suppressed, enabling TG2 to participate in alternative scaffolding and signaling functions [39,40,42]. Thus, the reduced transamidase activity observed in peMSCs despite increased TG2 expression is consistent with a predominantly non-transamidating functional state and raises the possibility that alternative scaffolding and signaling functions of TG2 become more prominent, although the conformational and nucleotide-binding state of TG2 was not directly examined in the present study. Similarly, the upstream signaling mechanisms responsible for the sustained upregulation of TG2 in endometriosis-derived eMSCs were not investigated in the present study. Given the complex microenvironment of endometriosis, identifying the extracellular cues and intracellular signaling pathways that regulate TG2 expression represents an important direction for future investigation. The biological implications of this altered TG2 functional profile may be particularly relevant to the disease-associated behavior of peMSCs. Whereas transamidase-active TG2 contributes to protein cross-linking and ECM stabilization, non-transamidating TG2 can function as a matricellular and cell-surface scaffold through interactions with extracellular matrix components and adhesion receptors [14,43,44,45]. Consistent with this model, the present study demonstrated markedly enhanced associations of TG2 with ITGβ-1 and SDC-4 in peMSCs, despite comparable overall levels of these receptors in heMSCs and peMSCs. These findings suggest that the increased abundance of TG2 in peMSCs may promote its membrane-associated interactions with adhesion receptors rather than simply enhancing its transamidase activity. Such a mechanism could facilitate adhesion-dependent signaling and cytoskeletal regulation, providing a potential molecular basis for the enhanced migratory and invasive phenotype observed in endometriosis-derived eMSCs [14,28,43,44,45].

The biological significance of these enhanced membrane-associated interactions is supported by previous studies demonstrating that TG2 functions as a multifunctional extracellular scaffold capable of interacting with fibronectin, integrins, and syndecans [14,43,44]. Through these interactions, TG2 contributes to focal adhesion assembly, cytoskeletal organization, and bidirectional signaling between the extracellular matrix and the actin cytoskeleton [19,43]. We have previously demonstrated that TG2 cooperates with fibronectin, β1-integrins, and syndecan-4 to regulate stable cell adhesion and migration [14]. More recently, TG2 has been shown to facilitate integrin- and syndecan-mediated activation of the small GTPases RhoA and Rac1, thereby regulating cytoskeletal dynamics, cell adhesion, spreading, and migration [46]. Collectively, these findings support a role for TG2 as an organizer of adhesion-dependent signaling in addition to its classical ECM cross-linking activity [21]. Consistent with this molecular framework, peMSCs exhibited markedly enhanced migratory capacity, whereas TG2 downregulation substantially reduced migration to levels comparable with those observed in heMSCs. These findings provide functional evidence that elevated TG2 expression contributes to the enhanced migratory phenotype of endometriosis-derived eMSCs. This may be particularly relevant to ectopic lesion establishment, which requires displaced endometrial cells not only to reach the peritoneal cavity but also to adhere to ectopic surfaces and subsequently migrate within the surrounding tissue microenvironment [5]. Thus, enhanced TG2-dependent adhesion signaling may provide one mechanism by which endometriosis-derived stromal cells acquire the migratory properties required for ectopic implantation and lesion establishment [5,14,43]. Beyond migration, successful lesion establishment requires cells to penetrate tissue barriers and remodel the surrounding extracellular matrix. Consistent with this requirement, peMSCs exhibited substantially greater invasive capacity than heMSCs, whereas TG2 downregulation markedly suppressed this phenotype. Together, these findings indicate that TG2 contributes to both the enhanced migratory and invasive behavior of endometriosis-derived eMSCs, supporting a regulatory role for TG2 in cellular processes that may facilitate ectopic lesion establishment.

The enhanced invasive phenotype observed in peMSCs may, at least in part, reflect increased extracellular matrix remodeling. Matrix metalloproteinases, particularly MMP-2 and MMP-9, are well-established mediators of basement membrane degradation, extracellular matrix turnover, and tissue invasion, and dysregulated MMP activity has been implicated in endometriotic lesion establishment and progression [11,28,47]. Consistent with this framework, peMSCs exhibited increased MMP-2 and MMP-9 expression and gelatinolytic activity, whereas TG2 silencing markedly attenuated both responses. These findings suggest that TG2 contributes to the invasive phenotype of endometriosis-derived eMSCs not only through its association with cell-surface adhesion receptors but also through regulation of MMP-dependent matrix remodeling. This interpretation is supported by previous work demonstrating that alterations in TG2 functional state can influence MMP-2 and MMP-9 activation and cell migration [28]. Collectively, the present findings support a model in which TG2 links adhesion-associated signaling with extracellular matrix remodeling to facilitate the invasive behavior of peMSCs.

The increased clonogenic capacity of peMSCs provides an additional dimension to the disease-associated phenotype observed in this study. Colony-forming ability is widely recognized as a functional indicator of mesenchymal stem/progenitor cells and reflects their capacity for clonal expansion and sustained proliferation [8,38,48]. The marked reduction in clonogenicity following TG2 silencing suggests that TG2 contributes not only to the enhanced migratory and invasive behavior of peMSCs but also to the maintenance of their clonogenic potential. This finding is particularly relevant in the context of the increased expression of the perivascular/progenitor-associated markers CD146, W5C5/SUSD2, and PDGFR observed in peMSCs [29,37,38]. Together, the enhanced clonogenic capacity and progenitor-associated immunophenotype support the presence of a highly plastic stromal population capable of sustained expansion while retaining enhanced migratory and invasive properties. Such cellular characteristics may facilitate the establishment, expansion, and long-term persistence of ectopic lesions, processes central to endometriosis progression [11,48,49].

Beyond its role in regulating adhesion, migration, and invasion, TG2 also appears to contribute to the enhanced proliferative capacity of endometriosis-derived mesenchymal stem cells. Sustained lesion growth requires not only successful implantation but also continuous cellular expansion following establishment at ectopic sites [5,11,48,49]. Consistent with this concept, peMSCs exhibited significantly increased proliferative activity accompanied by molecular alterations favoring cell-cycle progression. Increased expression of Cyclin D3, CDK4, Cyclin E1, and CDK2, together with reduced p27 expression, was associated with a marked redistribution of cells from the G0/G1 to the S phase, supporting accelerated G1/S progression and reduced inhibitory control of the cell cycle [50,51,52]. Importantly, TG2 downregulation reversed these molecular alterations, restored G0/G1 accumulation, reduced the S-phase population, and suppressed the enhanced proliferative phenotype of peMSCs. These findings identify TG2 as an important contributor to the dysregulated cell-cycle progression associated with endometriosis-derived eMSCs. The mechanisms linking TG2 to cell-cycle regulation are likely multifactorial. TG2-mediated interactions with extracellular matrix components and cell-surface adhesion receptors have been implicated in signaling pathways that regulate cell survival and proliferation [14,19,28,53]. Such functions are particularly relevant to endometriosis, where displaced endometrial-derived cells must survive and expand within an ectopic microenvironment to establish persistent lesions [54]. Notably, TG2 silencing was accompanied by reduced PDGFR expression and coordinated downregulation of the Cyclin D3/CDK4 and Cyclin E1/CDK2 axes, together with restoration of p27 expression. Although a direct mechanistic link between TG2-associated cell-surface signaling and these cell-cycle regulators remains to be established, these coordinated changes suggest that TG2 may contribute to signaling networks that couple the disease-associated surface phenotype of peMSCs to their enhanced proliferative capacity.

Among all findings presented in this study, the shRNA-mediated silencing experiments provide the strongest evidence that TG2 contributes directly to the disease-associated phenotype of peMSCs rather than serving solely as a disease-associated marker. TG2 downregulation simultaneously reduced the expression of the perivascular/progenitor-associated markers CD146, W5C5/SUSD2, and PDGFR and markedly attenuated clonogenicity, migration, invasion, MMP-2 and MMP-9 expression and activity, proliferation, and dysregulated cell-cycle progression. Importantly, canonical MSC markers remained largely unchanged following TG2 downregulation, while hematopoietic and endothelial markers remained negligible. Thus, suppression of TG2 selectively reversed multiple disease-associated characteristics without disrupting the fundamental mesenchymal immunophenotype of peMSCs. These findings identify TG2 as a potential therapeutic target for modulating pathological cellular behaviors associated with endometriosis while preserving the core phenotypic characteristics of endometrial MSCs. The present findings may also help reconcile the diverse and context-dependent biological functions attributed to TG2. In inflammatory and fibrotic tissues, transamidase-active TG2 can promote extracellular matrix cross-linking and stabilization, thereby contributing to tissue remodeling and fibrosis [43]. In contrast, the present data suggest that, in endometriosis-derived eMSCs, increased TG2 expression occurs despite reduced transamidase activity and is accompanied by enhanced associations with ITGβ-1 and SDC-4, supporting a prominent role for non-transamidating adhesion- and signaling-related functions [14,28,55]. Rather than representing contradictory activities, these observations are consistent with the ability of TG2 to adopt distinct functional states depending on its biochemical and cellular microenvironment [39,40]. It is therefore conceivable that the relative contribution of the transamidase and non-transamidating functions of TG2 may vary according to the cellular context and potentially across different stages of endometriosis progression. Whether TG2 contributes predominantly to adhesion, migration, and invasion during early lesion establishment and subsequently participates in extracellular matrix stabilization and fibrosis through increased transamidase activity remains an intriguing possibility that warrants direct investigation in stage-specific and in vivo models. A limitation of the present study is that eMSCs were established using a non-enzymatic outgrowth method rather than prospectively enriched from fresh endometrial tissue using eMSC-associated markers such as SUSD2/W5C5 or CD146/PDGFR. However, the non-enzymatic isolation approach used in the present study has been previously established and characterized by our group [29,56], and the resulting cultures were comprehensively characterized for canonical mesenchymal, hematopoietic/endothelial, and eMSC/perivascular-associated markers. Nevertheless, future studies employing prospective enrichment may help further define the specific eMSC subpopulations associated with TG2-dependent phenotypes. At the same time, the present primary-cell model enables the cell-level contribution of TG2 to the endometriosis-derived eMSC phenotype to be investigated under controlled experimental conditions. However, culture and cellular expansion cannot fully reproduce the complex cellular and extracellular microenvironment of an intact endometriotic lesion. A further limitation of the present study is that TG2 expression was evaluated in isolated primary eMSCs rather than directly in intact eutopic endometrium or ectopic endometriotic lesions. Validation of these findings at the tissue level, including the spatial localization of TG2 within endometriotic lesions, represents an important direction for future studies. Future biomarker-oriented validation studies should additionally include appropriate gynecological disease-control groups, formal assessment of diagnostic performance, including sensitivity and specificity, and evaluation of clinically feasible sampling and assay strategies.

Although potential interindividual variability was minimized by standardizing tissue collection conditions and using independently derived primary cell preparations, the relatively limited number of donors remains a limitation of the present study. Given the clinical and molecular heterogeneity of endometriosis, validation in larger and clinically more diverse independent cohorts will be important. Moreover, the present cohort was primarily sensitive to very large between-group effects, and small-to-moderate differences may therefore have remained undetected. These considerations should be taken into account when interpreting the generalizability of the present findings.

Collectively, the present study supports a model in which TG2 contributes to multiple interconnected features of the disease-associated peMSC phenotype. In contrast to heMSCs, peMSCs exhibited markedly increased TG2 expression despite reduced Ca^2+^-dependent transamidase activity, together with enhanced associations of TG2 with the cell-surface adhesion receptors ITGβ-1 and SDC-4. This altered functional profile was accompanied by increased expression of perivascular/progenitor-associated markers, enhanced clonogenicity, migration and invasion, increased MMP-2 and MMP-9 expression and activity, and dysregulated proliferation and cell-cycle progression. Importantly, TG2 downregulation simultaneously reversed these disease-associated characteristics while preserving the fundamental mesenchymal immunophenotype of the cells, providing strong functional evidence that TG2 contributes to the coordinated regulation of the endometriosis-associated peMSC phenotype. These findings further suggest that the non-transamidating functions of TG2, including its interactions with extracellular matrix components and cell-surface adhesion receptors, may represent therapeutically relevant mechanisms in endometriosis. Accordingly, strategies aimed at modulating TG2 scaffold functions or disrupting specific TG2-mediated interactions with fibronectin, ITGβ-1, and SDC-4 warrant further investigation as potential approaches to suppress disease-associated cellular behaviors beyond those achievable through inhibition of TG2 transamidase activity alone [14,16,28].

## 4. Materials and Methods

### 4.1. Human Tissue Samples, Isolation and Characterization of eMSCs

Human endometrial tissue samples were obtained from five patients with endometriosis and five healthy volunteers after written informed consent. The demographic and clinical characteristics of the study participants are provided in Supplementary Table S1. The study protocol was approved by the Yeditepe University Human Research Ethics Committee (Approval No. 63/509) and was conducted in accordance with the Declaration of Helsinki. Patient selection was based on the criteria established by the American Society for Reproductive Medicine (ASRM).

eMSCs were isolated from endometrial biopsy specimens obtained from five healthy volunteers (heMSCs) and five patients with endometriosis (peMSCs), as previously described by our group [29,56]. All endometrial biopsy specimens were collected during the proliferative phase of the menstrual cycle, thereby minimizing potential variability associated with cycle-dependent changes in the endometrium. None of the participants were receiving hormonal treatment at the time of tissue collection. Briefly, tissue samples were washed with PBS) containing antibiotics, minced into small fragments, and directly cultured using the non-enzymatic isolation method. The isolated cells were maintained in low-glucose DMEM supplemented with 20% FBS until approximately 80% confluence. Cells derived from each donor were maintained and analyzed individually throughout the study without pooling. The isolated cells were characterized by flow cytometry as previously described [29,56]. eMSCs were identified by the expression of CD146 (Abcam, Cambridge, UK; Cat. No. ab209298; 1:1000), PDGFR (Abcam, Cambridge, UK; Cat. No. ab99896; 1:1000), W5C5 (BioLegend, San Diego, CA, USA; Cat. No. 327406; 1:1000), CD44 (Abcam, Cambridge, UK; Cat. No. ab269300; 1:1000), CD29 (Abcam, Cambridge, UK; Cat. No. ab218273; 1:1000), CD73 (Abcam, Cambridge, UK; Cat. No. ab157335; 1:1000), ITGβ-1 (Abcam, Cambridge, UK; Cat. No. ab7168; 1:1000), CD90 (Abcam, Cambridge, UK; Cat. No. ab95700, 1:1000) and CD105 (Abcam, Cambridge, UK; Cat. No. ab314277; 1:1000) while the absence of CD14 (Abcam, Cambridge, UK; Cat. No. ab28061; 1:1000), CD31 (Abcam, Cambridge, UK; Cat. No. ab27333; 1:1000), CD34 (Abcam, Cambridge, UK; Cat. No. ab131589; 1:1000) and CD45 (BioLegend, San Diego, CA, USA; Cat. No. 304008; 1:2000) confirmed the lack of hematopoietic cell contamination. For flow-cytometric analysis, gating thresholds were established using the corresponding mouse IgG-FITC and IgG-PE isotype controls. All experiments were performed independently using cells from each donor, resulting in five independent biological replicates per experimental group. All experiments were performed using passage 3 cells, and cells were not used beyond passage 5. Cultures were routinely screened for mycoplasma contamination by PCR, with no contamination detected during routine monitoring. Cell viability was assessed by Trypan Blue exclusion during cell counting prior to experimental seeding.

### 4.2. TG2 Expression and Activity Analysis

TG2 transamidase activity was determined using the biotinylated cadaverine (BTC) incorporation assay as previously described [24]. Briefly, 96-well plates were coated with 10 μg/mL human plasma fibronectin (Sigma-Aldrich, Burlington, MA, USA) overnight at 4 °C and blocked with 3% BSA. eMSCs were seeded onto fibronectin-coated plates at a density of 20,000 cells/well in serum-free DMEM containing 0.132 mM biotinylated cadaverine (BTC; Biotium, USA). Fibronectin-coated wells containing serum-free medium and BTC without cells served as background controls. Following incubation for 3 h at 37 °C, BTC incorporation was detected using extravidin-horseradish peroxidase (HRP; Sigma-Aldrich, Burlington, MA, USA), and absorbance was measured at 450 nm using a microplate reader. TG2 gene expression was determined by RT-qPCR as previously described by our group [28]. Total RNA was isolated from heMSCs and peMSCs using peqGOLD TriFast reagent (PEQLAB, Erlangen, Germany), and cDNA was synthesized using the Sensiscript Reverse Transcriptase Kit (Qiagen, Hilden, Germany) according to the manufacturer’s instructions. Quantitative PCR was performed using the QuantiTect SYBR Green PCR Kit (QIAGEN, Hilden, Germany; Cat. No. 204143)) and TG2-specific primers (QIAGEN, Hilden, Germany; GeneGlobe ID: PPH23362A; Cat. No. 330001). Relative gene expression levels were calculated after normalization to the housekeeping gene 18S rRNA (QIAGEN, Hilden, Germany; GeneGlobe ID: QT00199367; Cat. No. 249900).

### 4.3. Western Blot and Co-Immunoprecipitation Analyses

Protein expression levels of TG2 (Invitrogen/Thermo Fisher Scientific, Waltham, MA, USA; Cat. No. MA5-12739; 1:200), integrin β-1 (ITGβ-1) ((Invitrogen/Thermo Fisher Scientific, Waltham, MA, USA; Cat. No. A303-735A; 1:10,000), syndecan-4 (SDC-4) (Invitrogen/Thermo Fisher Scientific, Waltham, MA, USA; Cat. No. 36-3100; 1:10,000), Cyclin E1 (Invitrogen/Thermo Fisher Scientific, Waltham, MA, USA; Cat. No. MA5-14336; 1:10,000), cyclin-dependent kinase 2 (CDK2) (Invitrogen/Thermo Fisher Scientific, Waltham, MA, USA; Cat. No. MA5-17052; 1:2000), Cyclin D3 (Cell Signaling Technology, Danvers, MA, USA; Cat. No. 2936; 1:2000), CDK4 (Cell Signaling Technology, Danvers, MA, USA; Cat. No. 12790; 1:1000), and p27 (Cell Signaling Technology, Danvers, MA, USA; Cat. No. 3688; 1:1000) were determined by Western blot analysis as previously described by our group [28,57]. Briefly, total cellular proteins were extracted using RIPA lysis buffer (Thermo Fisher Scientific, Waltham, MA, USA, Cat. No. 89900) and protein concentrations were determined by the Lowry assay. Equal amounts of protein (50 μg per lane) were separated by 8–12% SDS-PAGE and transferred onto either 0.45 μm or 0.22 μm nitrocellulose membranes, depending on the molecular weight of the target protein. Membranes were incubated overnight at 4 °C with the appropriate primary antibodies, followed by horseradish peroxidase (HRP)-conjugated secondary antibodies for 2 h at room temperature. Immunoreactive bands were visualized using enhanced chemiluminescence (ECL) and imaged with the ChemiDoc XRS+ Imaging System (Bio-Rad Laboratories, Hercules, CA, USA). β-Actin was used as the loading control.

Protein–protein interactions between TG2 and integrin β-1 or syndecan-4 were investigated by co-immunoprecipitation of isolated membrane fractions, as previously described [28]. Briefly, membrane fractions were pre-cleared by incubation with 1 µg mouse IgG for 1 h at 4 °C, followed by incubation with Protein G-coated Sepharose beads for 1 h to remove proteins binding non-specifically to mouse IgG. Following centrifugation, the pre-cleared samples were incubated with mouse anti-integrin β-1 (0.5 µg/mL; Santa Cruz Biotechnology, Cat. No. sc-8978, Dallas, TX, USA) or mouse anti-syndecan-4 (1.25 µg/mL; Abcam, Cat. No. ab24511, Cambridge, UK) antibodies for 1 h at 4 °C. Immune complexes were recovered using Protein G-coated Sepharose beads and analyzed by Western blotting using the TG2-specific CUB7402 antibody (Thermo Fisher Scientific, Cat. No. MA5-12739, Waltham, MA, USA).

### 4.4. Determination of MMP Expression and Activity

The expression levels of MMP-2 (QIAGEN, Hilden, Germany; GeneGlobe ID: QT00088396; Cat. No. 249900) and MMP-9 (QIAGEN, Hilden, Germany; GeneGlobe ID: QT00040040; Cat. No. 249900) were determined by RT-qPCR using the same experimental procedure described above for TG2 gene expression, with MMP-2- and MMP-9-specific primers. Relative gene expression levels were normalized to the housekeeping gene 18S rRNA. For MMP expression and activity analyses, equal numbers of cells were seeded in three technical replicate wells for each donor-derived culture and maintained in medium containing 2% FBS. After 48 h, conditioned media from the three technical replicate wells were collected and pooled, while the corresponding cells were used for RNA isolation and subsequent RT-qPCR analysis of MMP-2 and MMP-9 expression. The enzymatic activities of MMP-2 and MMP-9 were evaluated by gelatin zymography as previously described [28]. Equal volumes of the pooled conditioned media were centrifuged to remove detached cells and cellular debris and subsequently lyophilized. The recovered dried material was weighed, and equal amounts were reconstituted in loading buffer before electrophoresis. Briefly, conditioned media were subjected to SDS-PAGE containing 2% gelatin (type B) as a substrate under non-reducing conditions. Following electrophoresis, gels were incubated in renaturation and developing buffers, stained with Coomassie Brilliant Blue, and destained until clear lytic bands corresponding to gelatinase activity became visible. Gelatinolytic activity was quantified by densitometric analysis using ImageJ software.

### 4.5. Cell Proliferation and Cell Cycle Analysis

Cell proliferation and cell cycle distribution of heMSCs and peMSCs were evaluated using the WST-1 assay and the Cytell™ Cell Imaging System (GE Healthcare Life Sciences, Chicago, IL, USA), respectively, as previously described by our group [28]. Briefly, for the proliferation assay, eMSCs were seeded at 2000 cells/well in 96-well plates and cultured for 24, 48, 72, and 96 h. A calibration curve was established by seeding 2000, 5000, 10,000, 15,000, and 20,000 cells/well, and WST-1 absorbance was measured at 450 nm after incubation with the reagent according to the manufacturer’s instructions. For cell cycle analysis, eMSCs were seeded at 5 × 10^3^ cells/well and allowed to attach for 6 h before the culture medium was replaced with DMEM supplemented with 4% FBS for an additional 16 h. Cells were then stained with the Cytell™ Reactive G Cell Cycle reagent (GE Healthcare Life Sciences, Marlborough, MA, USA) for 45 min and analyzed using the Cell Cycle BioApp software Version 3.6.7.19 to determine the proportions of cells in the G0/G1, S, and G2/M phases [58].

### 4.6. Lentiviral shRNA-Mediated TG2 Silencing

TG2 expression was silenced in peMSCs using lentiviral shRNA particles (Santa Cruz Biotechnology, sc-37514-V, Dallas, TX, USA) while scrambled shRNA (Santa Cruz Biotechnology, sc-108080, Dallas, TX, USA) served as the negative control. According to the manufacturer, the TG2 shRNA lentiviral particles consist of a pool of three to five target-specific constructs, each encoding a 19–25 nt shRNA. The individual target sequences are not disclosed by the manufacturer. eMSCs were seeded and incubated overnight at 37 °C in a humidified atmosphere containing 5% CO_2_. Cells were pretreated with protamine sulfate in low-glucose DMEM for 12 h before transduction with TG2-specific shRNA lentiviral particles at a multiplicity of infection (MOI) of 5. Following 36 h of transduction, stable cell populations were selected using 3 μg/mL puromycin [59]. The efficiency of TG2 knockdown was confirmed by Western blot analysis and RT-qPCR.

### 4.7. Cell Migration, Invasion, and Colony Formation Assays

Cell migration was evaluated using a wound healing (scratch) assay, as described before [28]. Briefly, heMSCs and peMSCs were seeded in 24-well plates at a density of 3 × 10^5^ cells/well and incubated for 16 h in medium containing 2% FBS to allow cell attachment and formation of a confluent monolayer. A standardized linear scratch was then generated using a six-comb SPL Scratcher. Following scratch formation, the cells were maintained in medium containing 2% FBS throughout the 12 h wound-closure period. Wound closure was monitored at 0, 6, and 12 h using a Zeiss inverted microscope (Carl Zeiss, Oberkochen, Germany). The percentage of wound closure was quantified using ImageJ software (NIH, Bethesda, MD, USA).

The colony-forming properties of eMSCs were assessed by a colony-forming unit (CFU) assay as described [29,60]. For this purpose, 50 eMSCs were seeded onto matrix gel-coated 100 mm Petri dishes and maintained for 21 days. Colonies were fixed, stained with Giemsa G250 (Sigma-Aldrich, Saint Louis, MO, USA), and imaged using a Leica stereomicroscope (Leica Microsystems, Wetzlar, Germany).

Cell invasion was determined using Matrigel-coated Transwell inserts with 8 μm pore membranes (Corning, Glendale, AZ, USA) as described previously [28,61]. Briefly, equal numbers of cells (2.5 × 10^5^ cells) suspended in 200 μL serum-free DMEM were seeded into the upper chamber, while 750 μL complete medium was added to the lower chamber. After 18 h incubation at 37 °C in 5% CO_2_, non-invading cells were removed from the upper surface of the membrane. Invaded cells were fixed with 3.7% paraformaldehyde, permeabilized with 0.1% Triton X-100, stained with 0.5% crystal violet, and imaged using a Zeiss microscope (Carl Zeiss, Oberkochen, Germany). Images were acquired from five different fields of each insert, and the number of invading cells was quantified using ImageJ software (NIH, Bethesda, MD, USA).

### 4.8. Statistical Analysis

Each donor-derived eMSC preparation was considered an independent biological replicate (*n* = 5 per group). Although healthy donor-derived and endometriosis patient-derived samples are displayed using the same G1–G5 identifiers in the figures for presentation purposes, these identifiers do not denote matched healthy-patient pairs. The two groups comprise independent individuals and were analyzed as independent groups. Where assays were performed in three technical replicate wells, the measurements from the three wells were averaged within each donor-derived culture to obtain a single donor-level value before statistical analysis. Technical replicates were not treated as independent biological observations and did not increase the biological sample size. For SCR and shRNA experiments, the five donor-derived cultures remained the biological replicates, whereas SCR and shRNA-TG2 represented distinct experimental conditions applied to these cultures. Statistical analyses were performed using GraphPad Prism version 11.1 (GraphPad Software, San Diego, CA, USA). Data distribution was assessed prior to statistical analysis. Comparisons between two groups were performed using the unpaired Student’s *t*-test for normally distributed data or the Mann–Whitney U test for non-normally distributed data. Comparisons among multiple groups were performed using one-way analysis of variance (ANOVA), followed by the appropriate post hoc test. A *p* value < 0.05 was considered statistically significant. Because the study was exploratory and the number of independently derived primary human cultures was constrained, the sample size was not determined by an a priori power calculation. A sensitivity analysis was therefore performed using G*Power version 3.1.9.7 [3] to characterize the minimum standardized effect detectable for the independent heMSC versus peMSC comparison. Assuming a target power of 80% (1 − β = 0.80), a two-sided α of 0.05, equal group allocation, and *n* = 5 independent biological replicates per group, the minimum detectable standardized effect size was Cohen’s d = 2.02. After applying the small-sample correction, this corresponded to a Hedges’ g of approximately 1.83. Thus, under these assumptions, the present design had adequate sensitivity primarily for very large standardized differences between the two donor groups, whereas small-to-moderate effects could have remained undetected. Detailed statistical analysis results, including Hedges’ g effect-size estimates, are provided in Supplementary Tables S2–S4.

## 5. Conclusions

In conclusion, this study demonstrates that TG2 is not merely associated with endometriosis but contributes directly to the disease-associated phenotype of endometriosis-derived mesenchymal stem cells. Increased TG2 expression was consistently accompanied by enhanced stemness, migration, invasion, extracellular matrix remodeling, and proliferative signaling, whereas shRNA-mediated TG2 silencing markedly attenuated these pathological characteristics and shifted endometriosis-derived mesenchymal stem cells toward a phenotype resembling that of healthy controls. These findings identify TG2 as a functional contributor to the endometriosis-associated eMSC phenotype rather than a passive molecular alteration.

Importantly, the consistent overexpression of TG2 in patient-derived cells together with the profound phenotypic restoration achieved following its silencing highlights its translational potential as a disease-associated molecular marker warranting further evaluation for its diagnostic relevance and as a promising therapeutic target. The ability of TG2 suppression to simultaneously reverse multiple disease-associated processes further supports its potential as a therapeutic target in endometriosis.

Taken together, these findings provide a strong biological rationale for further evaluating TG2 as a candidate component of future biomarker panels for endometriosis. Furthermore, they support the development and preclinical evaluation of TG2-targeted therapeutic strategies—including selective TG2 inhibitors, gene-silencing approaches, or other TG2-modulating interventions—as potential therapeutic approaches for endometriosis. Future in vivo studies and clinical investigations will be essential to validate the diagnostic utility of TG2 and to determine whether therapeutic modulation of TG2 can be successfully translated into clinical management of endometriosis.

## 6. Patents

A patent application related to the findings reported in this manuscript has been published: “Novel Biological Markers in the Diagnosis of Endometriosis”, US Patent Application Publication No. US20240241134A1. The application relates to the use of tissue transglutaminase and associated biomarkers for the diagnosis of endometriosis in isolated endometrial mesenchymal stem cells. The application is currently listed as pending.

## Figures and Tables

**Figure 1:**
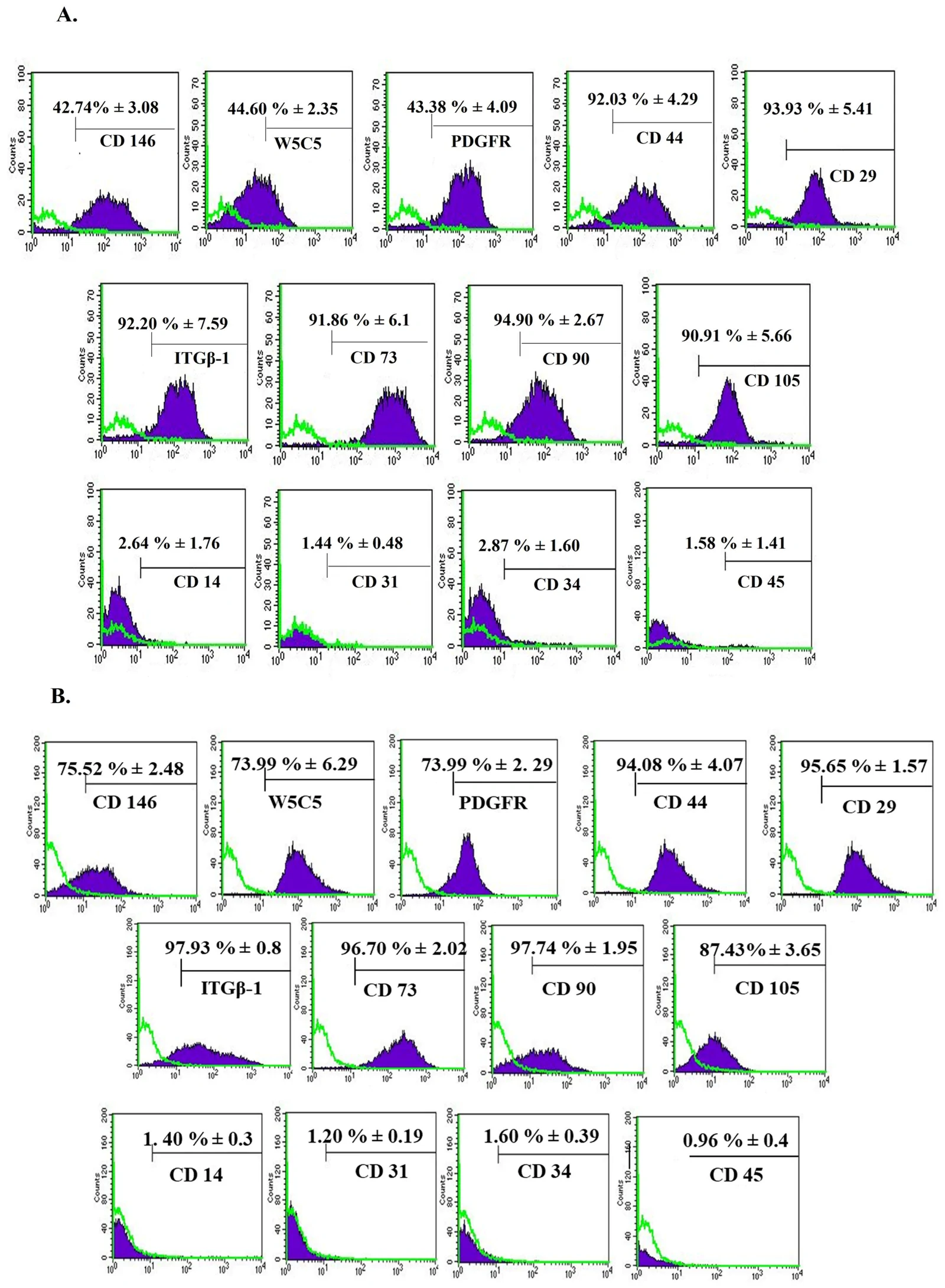
Characterization of endometrial mesenchymal stem cells (eMSCs) isolated from five healthy donors (heMSCs) and five patients with endometriosis (peMSCs) by flow cytometry. Representative histograms of healthy donor-derived eMSCs (**A**) and endometriosis-derived eMSCs (**B**) are shown. Purple-filled histograms represent staining with the indicated antibodies, whereas hollow green histograms represent the corresponding isotype IgG negative controls (NC). Quantitative analysis was performed using cells isolated from five independent donors per group, and the data are presented as mean ± SD.

**Figure 2:**
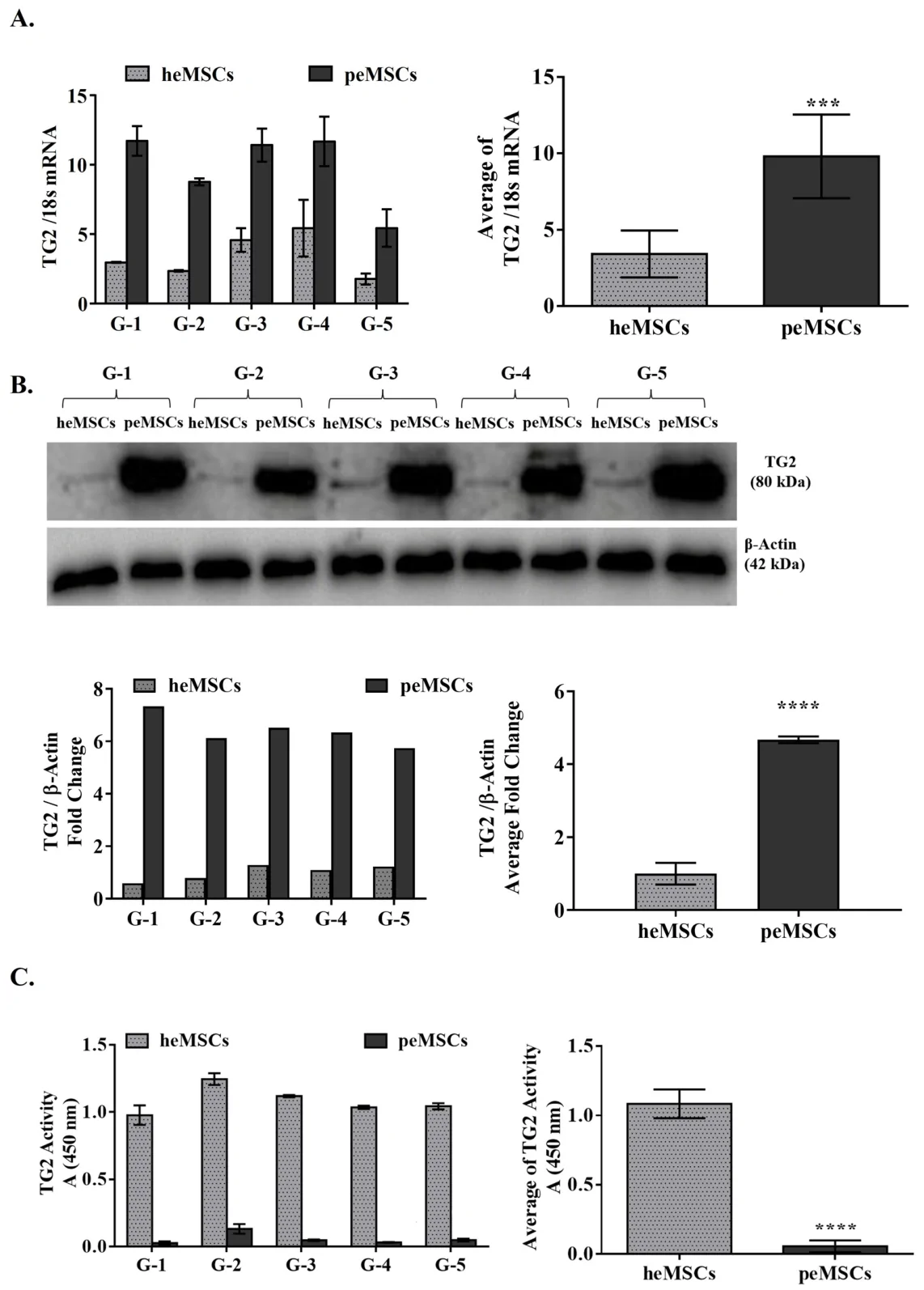
Comparative analysis of TG2 expression and transamidase activity in endometrial mesenchymal stem cells (eMSCs) isolated from five healthy donors (heMSCs) and five patients with endometriosis (peMSCs). Individual donor-derived samples are designated G1–G5. Each donor-derived sample represents one independent biological replicate, and statistical analysis was performed at the group level using *n* = 5 biological replicates per group. (**A**) TG2 mRNA expression was determined by RT-qPCR and normalized to 18S rRNA. Individual donor data (G1–G5) together with the corresponding group mean values are presented. (**B**) TG2 protein expression was evaluated by Western blot analysis. Representative immunoblots obtained from the five independent donor-derived samples in each group are shown, and densitometric analysis of TG2 protein expression was normalized to β-actin. (**C**) TG2 transamidase activity was determined using the biotin-X-cadaverine (BTC) incorporation assay. Individual donor values and the corresponding mean TG2 activities are presented. Data are expressed as mean ± SD. Statistical significance is indicated as *** *p* ≤ 0.001, and **** *p* ≤ 0.0001.

**Figure 3:**
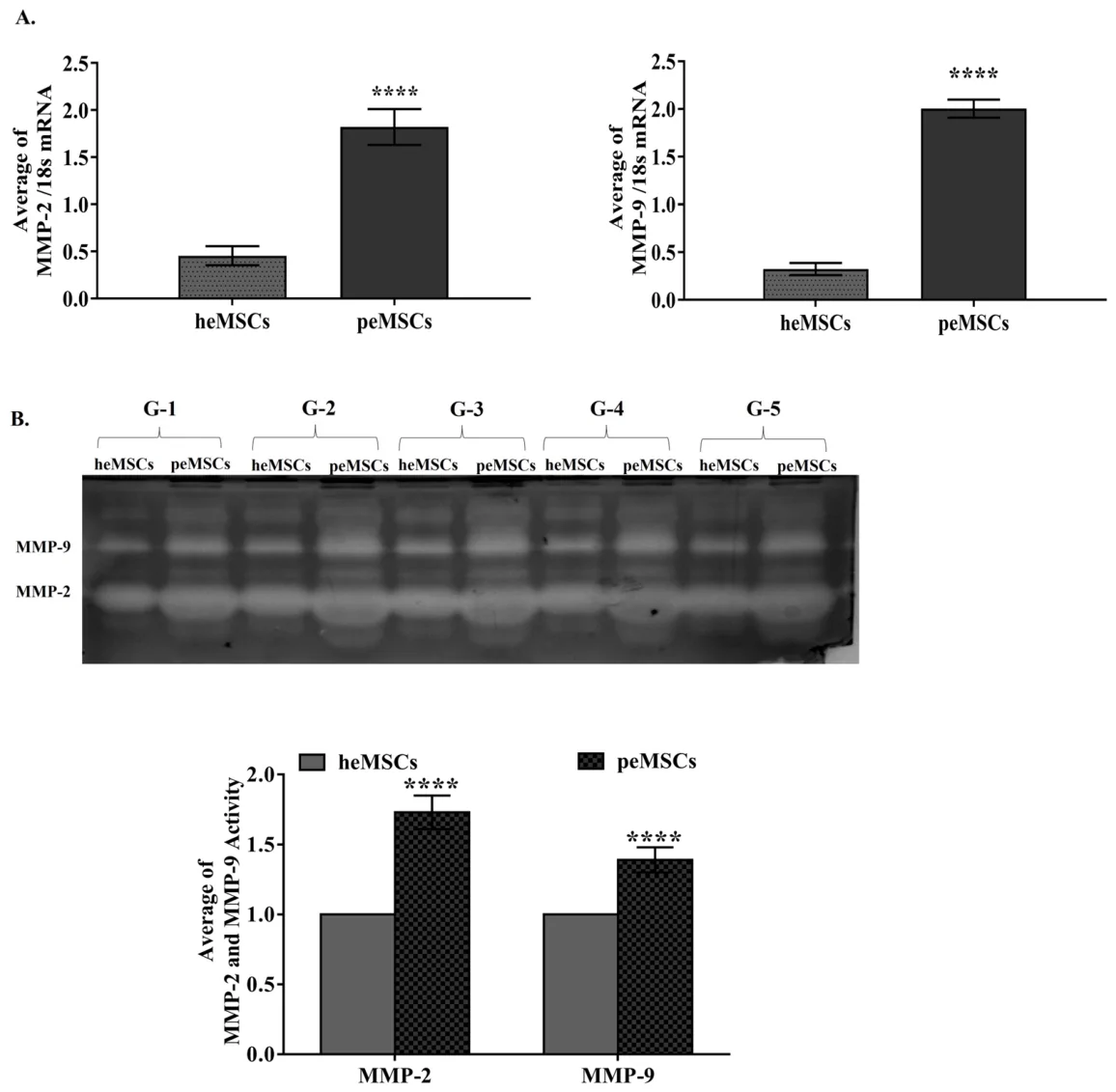
Analysis of MMP-2 and MMP-9 expression and enzymatic activity in eMSCs isolated from five healthy donors (heMSCs) and five patients with endometriosis (peMSCs). Individual donor-derived samples are designated G1–G5. (**A**) MMP-2 and MMP-9 mRNA expressions was determined by RT-qPCR and normalized to 18S rRNA. Individual donor data (G1–G5) together with the corresponding group mean values are presented. (**B**) MMP-2 and MMP-9 enzymatic activity was evaluated by zymography. Zymograms obtained from the five independent donor-derived samples in each group are shown, and densitometric analysis of the lytic bands was performed using ImageJ version 1.54p software. Data are presented as mean ± SD. Statistical significance is indicated as **** *p* ≤ 0.0001.

**Figure 4:**
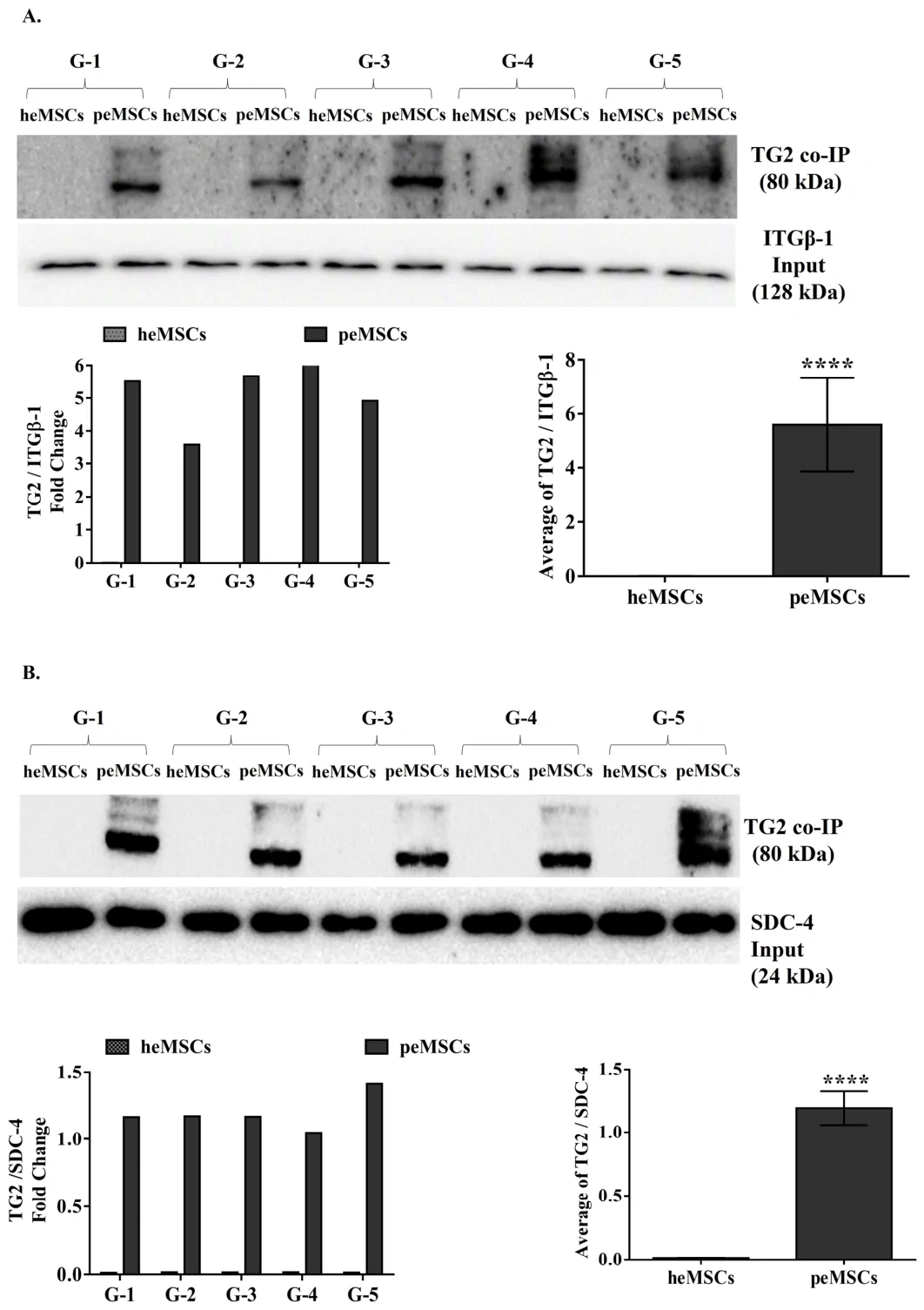
Analysis of TG2 association with integrin β-1 (ITGβ-1) and syndecan-4 (SDC-4) in eMSCs isolated from five healthy donors (heMSCs) and five patients with endometriosis (peMSCs). Co-immunoprecipitation analysis of (**A**) TG2–ITGβ-1 and (**B**) TG2–SDC-4 associations were performed using membrane fractions isolated from heMSCs and peMSCs. Western blot images of the input and immunoprecipitated (IP) fractions obtained from the five independent donor-derived samples in each group (G1–G5), together with the corresponding quantitative analyses, are shown. Input samples were analyzed for ITGβ-1 (**A**) and SDC-4 (**B**) to verify comparable levels of the respective immunoprecipitated proteins prior to immunoprecipitation. Data are presented as mean ± SD. Statistical significance is indicated as **** *p* ≤ 0.0001.

**Figure 5:**
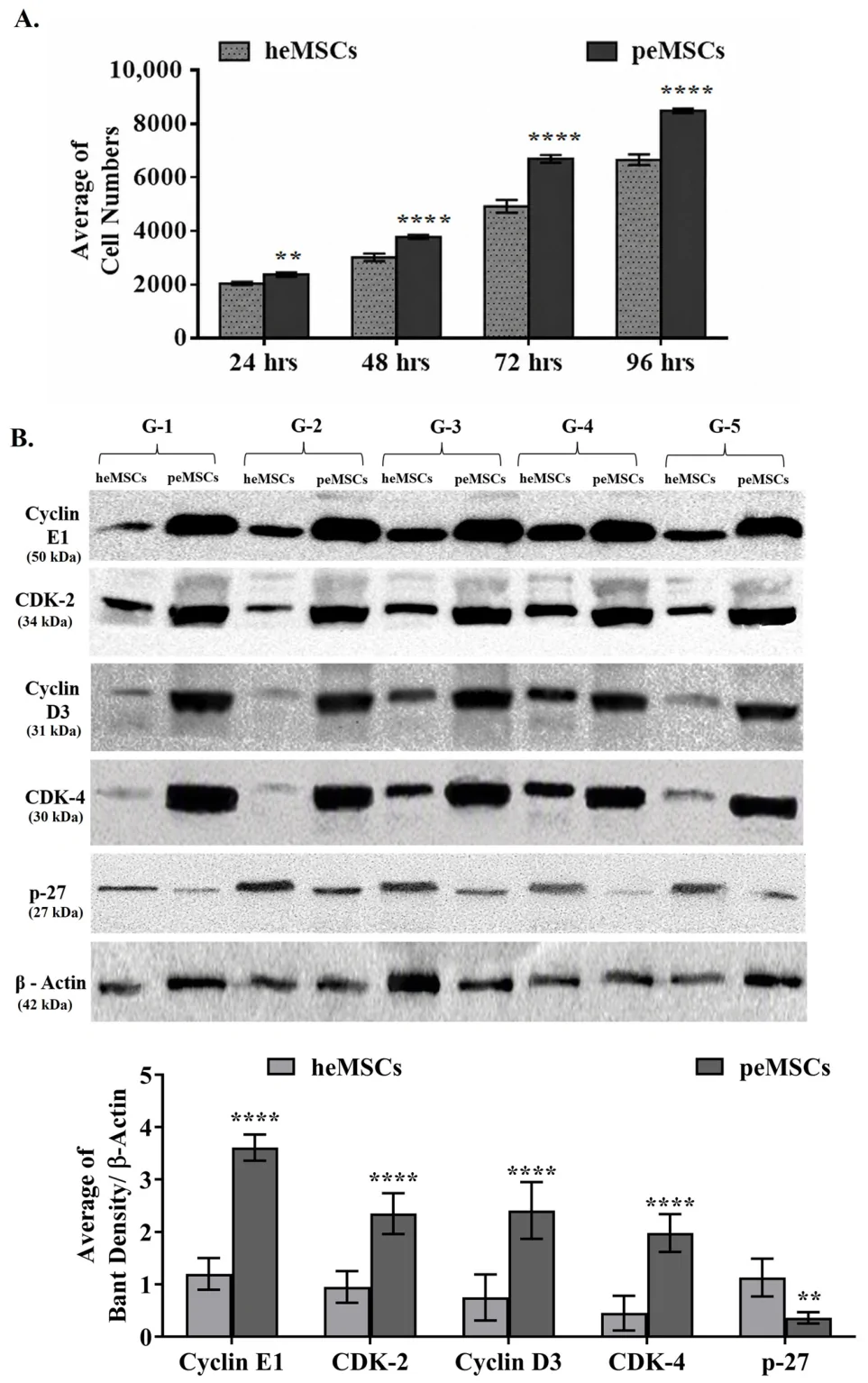
Analysis of cell proliferation and cell cycle regulatory proteins in eMSCs isolated from five healthy donors (heMSCs) and five patients with endometriosis (peMSCs). (**A**) Cell proliferation was evaluated by the WST-1 assay over a 96 h period. Individual donor data (G1–G5) together with the corresponding mean values are shown. (**B**) Western blot analysis of Cyclin E1, CDK2, Cyclin D3, CDK4, and p27 expression in five independent donor-derived samples in each group (G1–G5). Densitometric quantification of protein expression was performed after normalization to β-actin, and data are presented as mean ± SD. Statistical significance is indicated as ** *p* ≤ 0.01 and **** *p* ≤ 0.0001.

**Figure 6:**
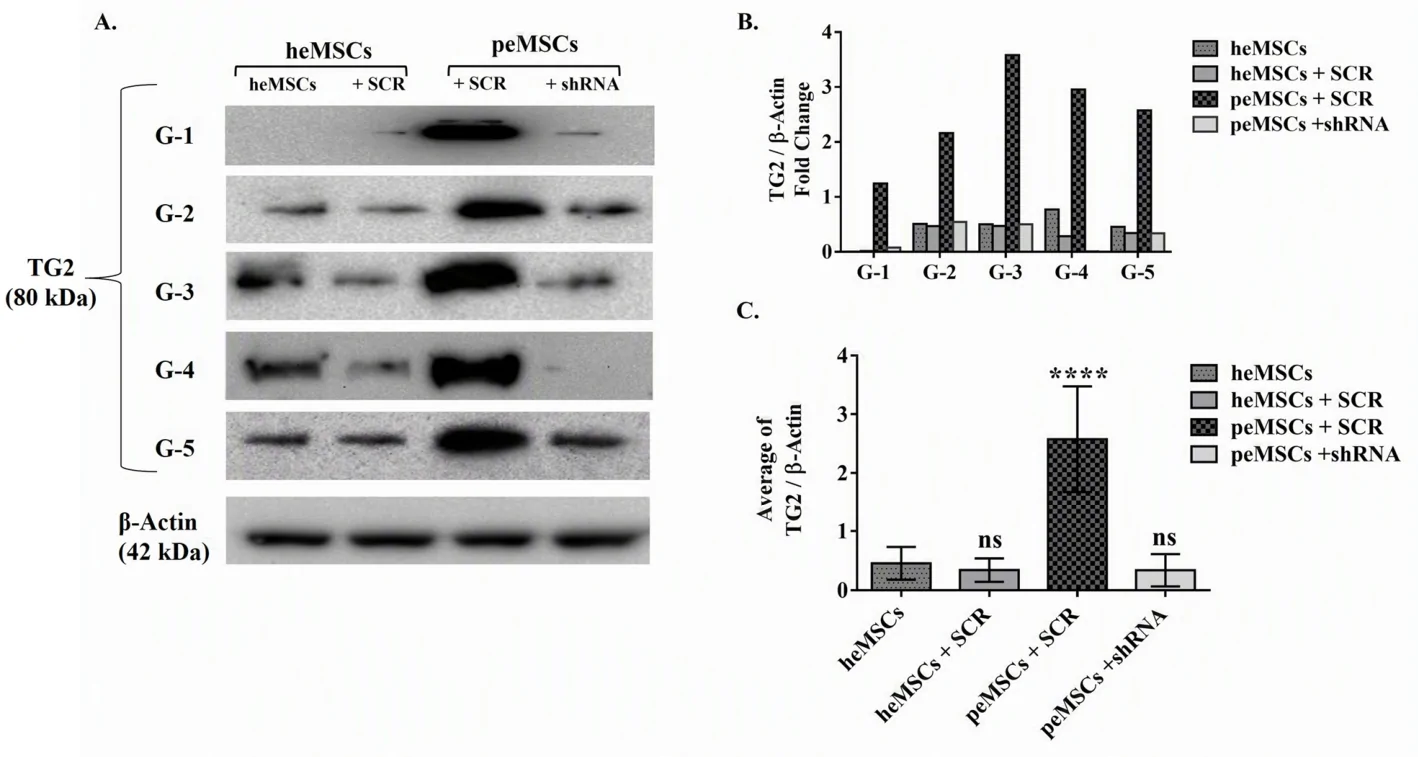
Analysis of TG2 protein expression following shRNA-mediated silencing in eMSCs isolated from five healthy donors (heMSCs) and five patients with endometriosis (peMSCs). (**A**) Western blot analysis of TG2 protein expression in untreated eMSCs and eMSCs transduced with either the scramble (SCR) control or TG2 shRNA from all five independent donor-derived samples in each group (G1–G5). (**B**) Individual densitometric analysis of TG2 protein expression normalized to β-actin for each donor-derived sample. (**C**) Comparison of the mean TG2/β-actin ratios among the four experimental groups. β-Actin was used as the loading control. Data are presented as mean ± SD. Statistical significance is indicated as ns (not significant) and **** *p* ≤ 0.0001.

**Figure 7:**
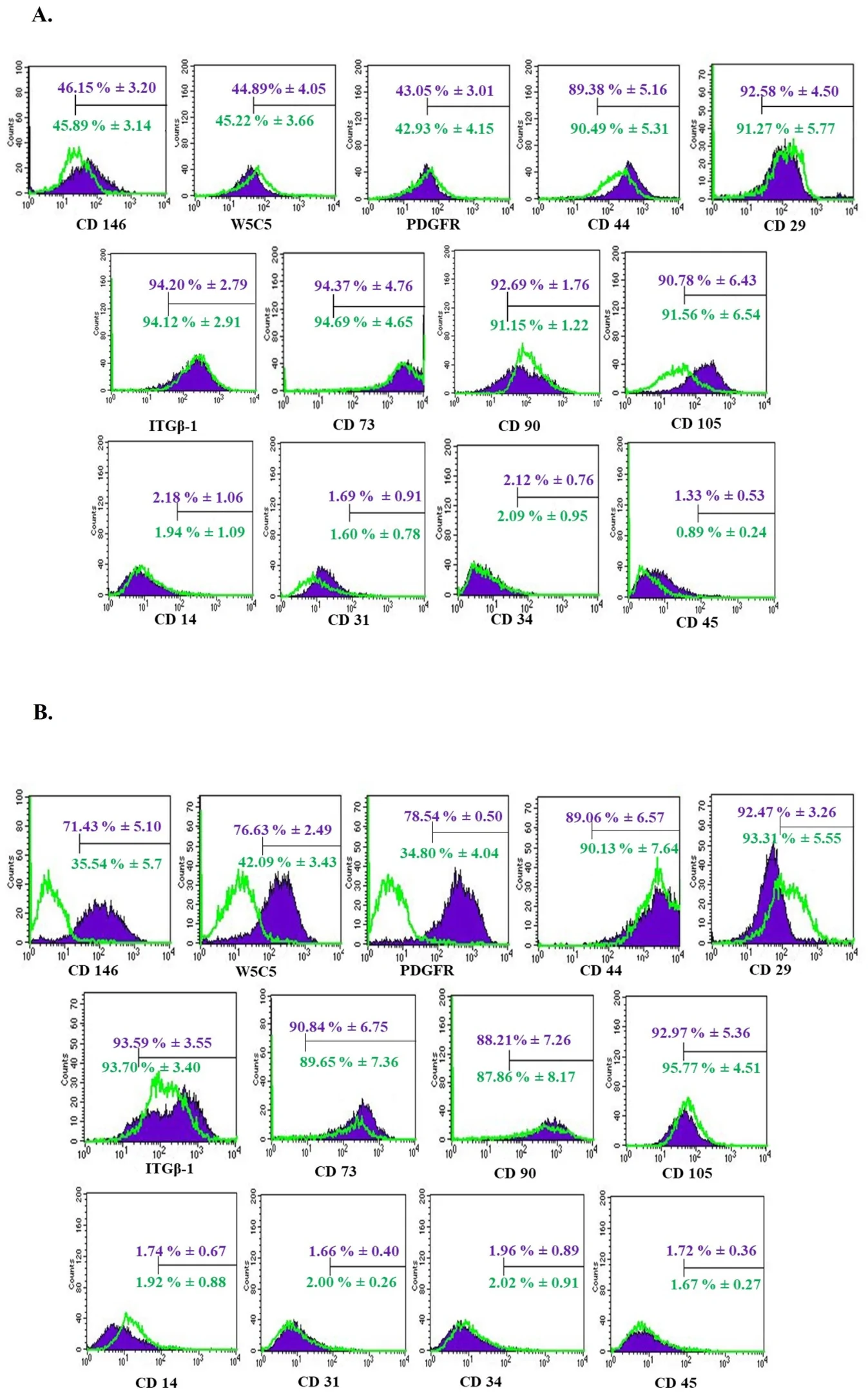
Surface marker expression in eMSCs following SCR and TG2 shRNA treatment. (**A**) Mean histograms of untreated heMSCs (purple) and heMSCs transduced with the scramble (SCR) control (green). (**B**) Mean histograms of peMSCs treated with the SCR control (purple) and TG2 shRNA (green). Quantitative analysis of surface marker expression is summarized in Table 2, and the corresponding individual histograms from the five independent donor-derived samples in each group (G1–G5) are presented in Supplementary Figures S4 and S5.

**Figure 8:**
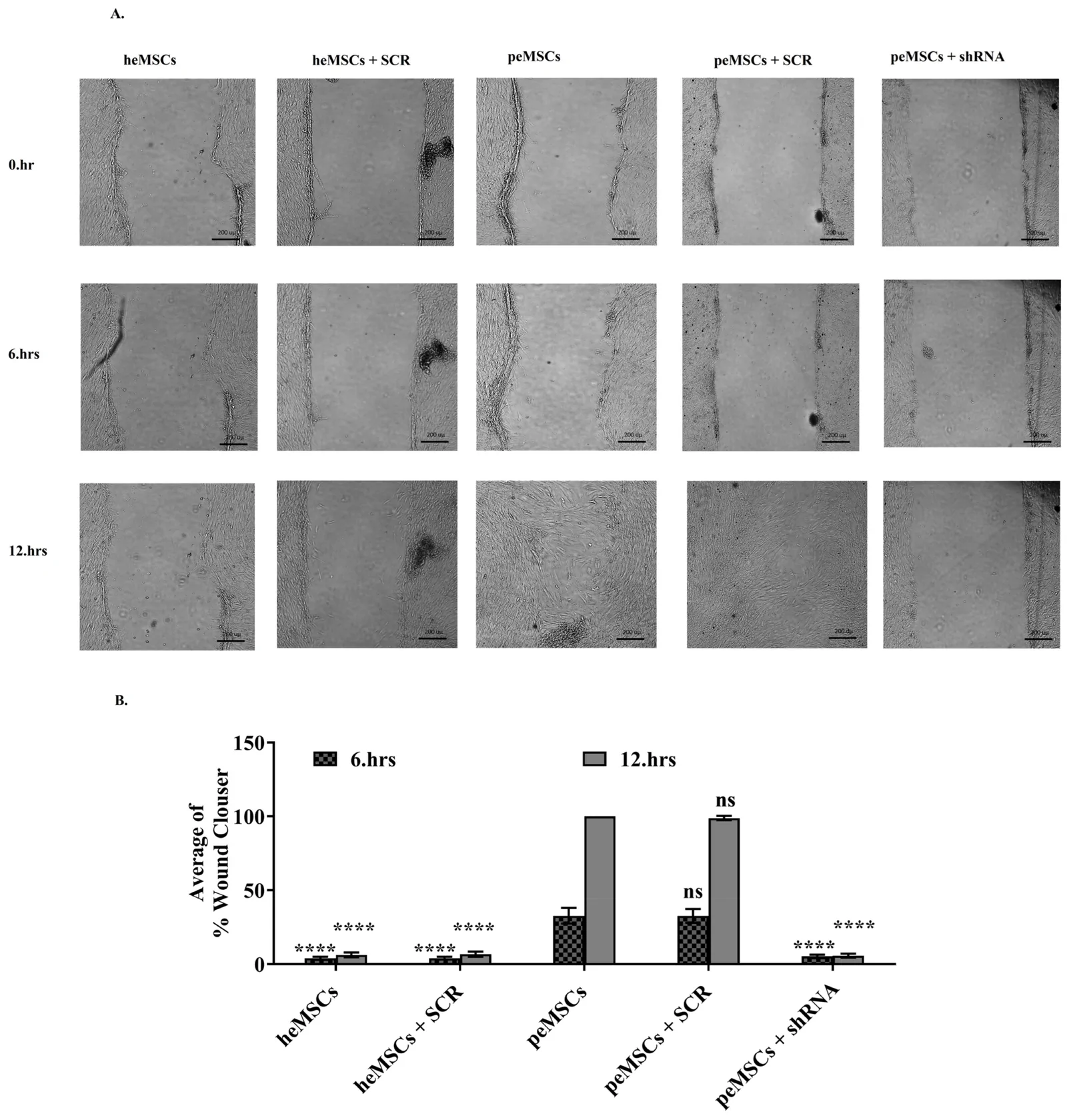
Analysis of cell migration following TG2 downregulation in eMSCs isolated from five healthy donors (heMSCs) and five patients with endometriosis (peMSCs). (**A**) Representative wound healing images obtained at 0, 6, and 12 h from all the five independent donor-derived samples in each group (G1–G5) under untreated conditions or following transduction with the scramble (SCR) control or TG2 shRNA. (**B**) Quantitative analysis of wound closure at 6 and 12 h. Data are presented as mean ± SD. The corresponding individual wound healing images for each sample are provided in Supplementary Figure S6. Statistical significance is indicated as ns (not significant) and **** *p* ≤ 0.0001.

**Figure 9:**
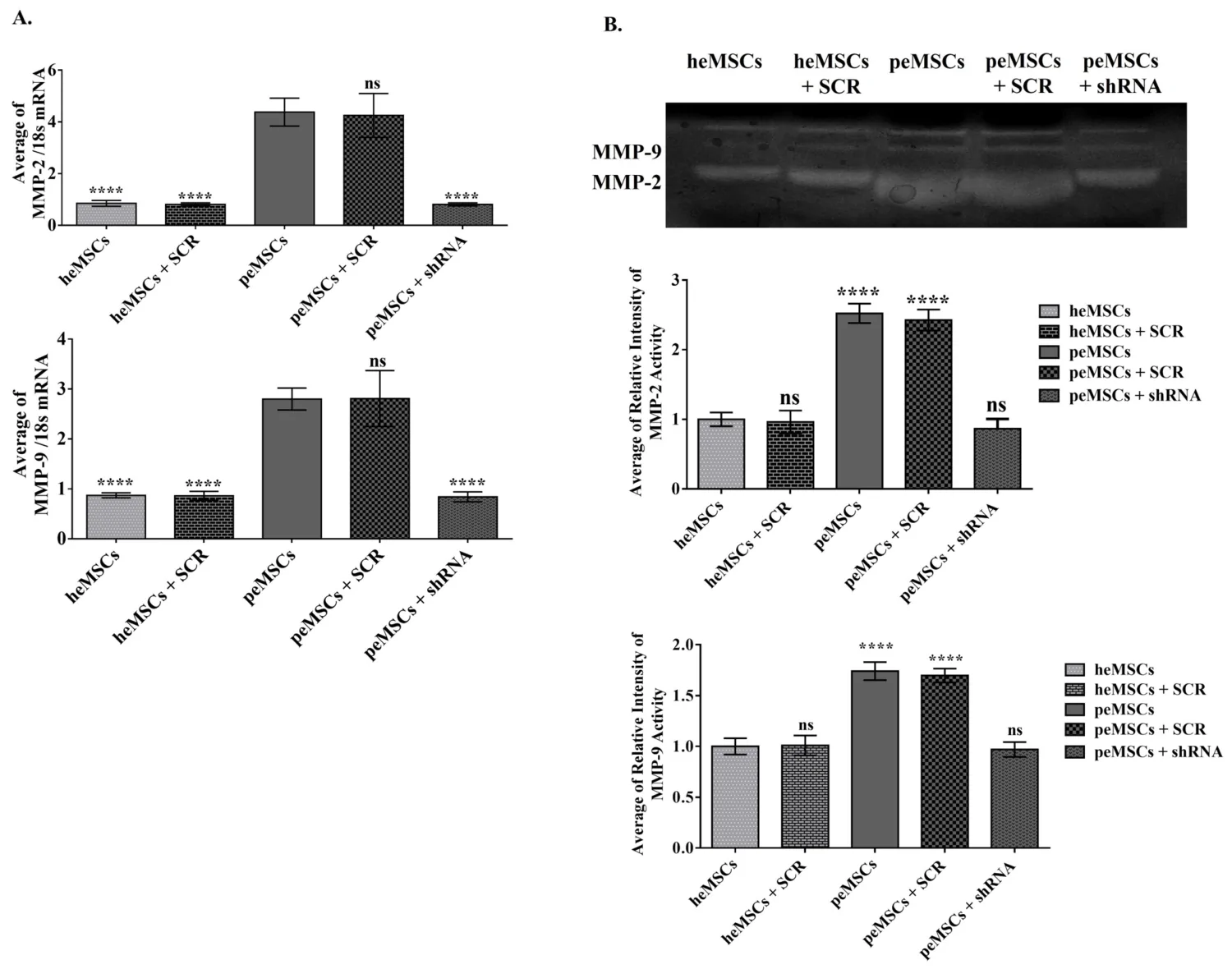
Analysis of MMP-2 and MMP-9 expressions and activity following TG2 downregulation in eMSCs isolated from five healthy donors (heMSCs) and five patients with endometriosis (peMSCs). (**A**) Relative MMP-2 and MMP-9 mRNA expression determined by RT-qPCR in untreated eMSCs and eMSCs transduced with either the scramble (SCR) control or TG2 shRNA. (**B**) Gelatin zymography analysis of MMP-2 and MMP-9 activity in the corresponding experimental groups, together with the quantitative analysis of gelatinolytic activity. Data are presented as mean ± SD. The corresponding individual gelatin zymograms from the five independent donor-derived samples in each group (G1–G5) are provided in Supplementary Figure S7. Statistical significance is indicated as ns (not significant) and **** *p* ≤ 0.0001.

**Figure 10:**
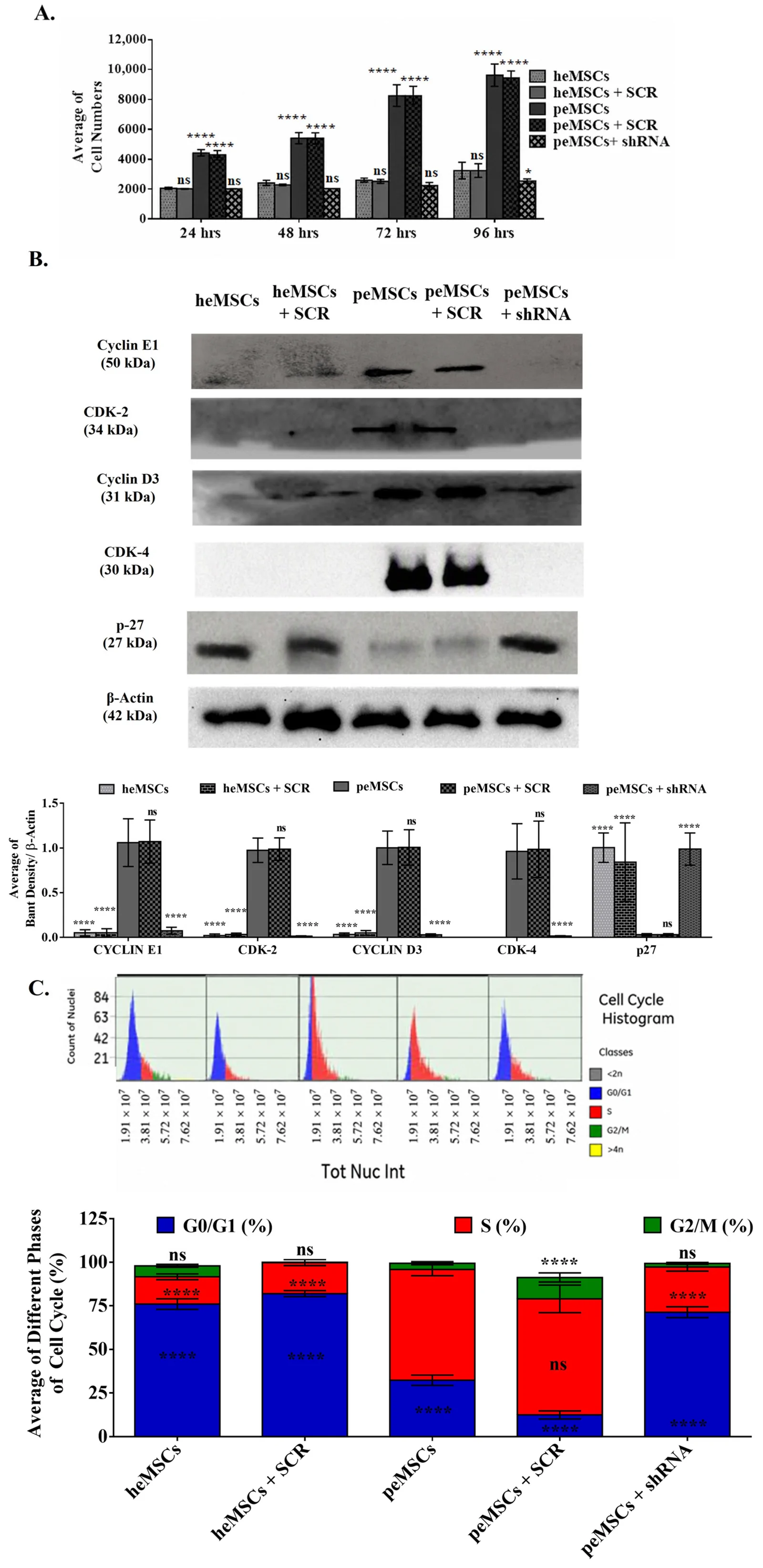
Analysis of cell proliferation and cell cycle progression following TG2 downregulation in eMSCs isolated from five healthy donors (heMSCs) and five patients with endometriosis (peMSCs). (**A**) Cell proliferation determined by WST-1 assay at 24, 48, 72, and 96 h in untreated eMSCs and eMSCs transduced with either the scramble (SCR) control or TG2 shRNA. (**B**) Western blot analysis of the cell cycle regulatory proteins Cyclin E1, CDK-2, Cyclin D3, CDK-4, and p27, together with the corresponding quantitative analysis. (**C**) Cell cycle distribution determined using the Cytell™ Cell Imaging System (GE Healthcare Life Sciences, Preston, UK), together with the corresponding quantitative analysis. Data are presented as mean ± SD. The corresponding individual Western blot images and cell cycle histograms obtained from the five independent donor-derived samples in each group (G1–G5) are provided in Supplementary Figures S8 and S9, respectively. Statistical significance is indicated as ns (not significant), * *p* ≤ 0.05 and **** *p* ≤ 0.0001.

**Figure 11:**
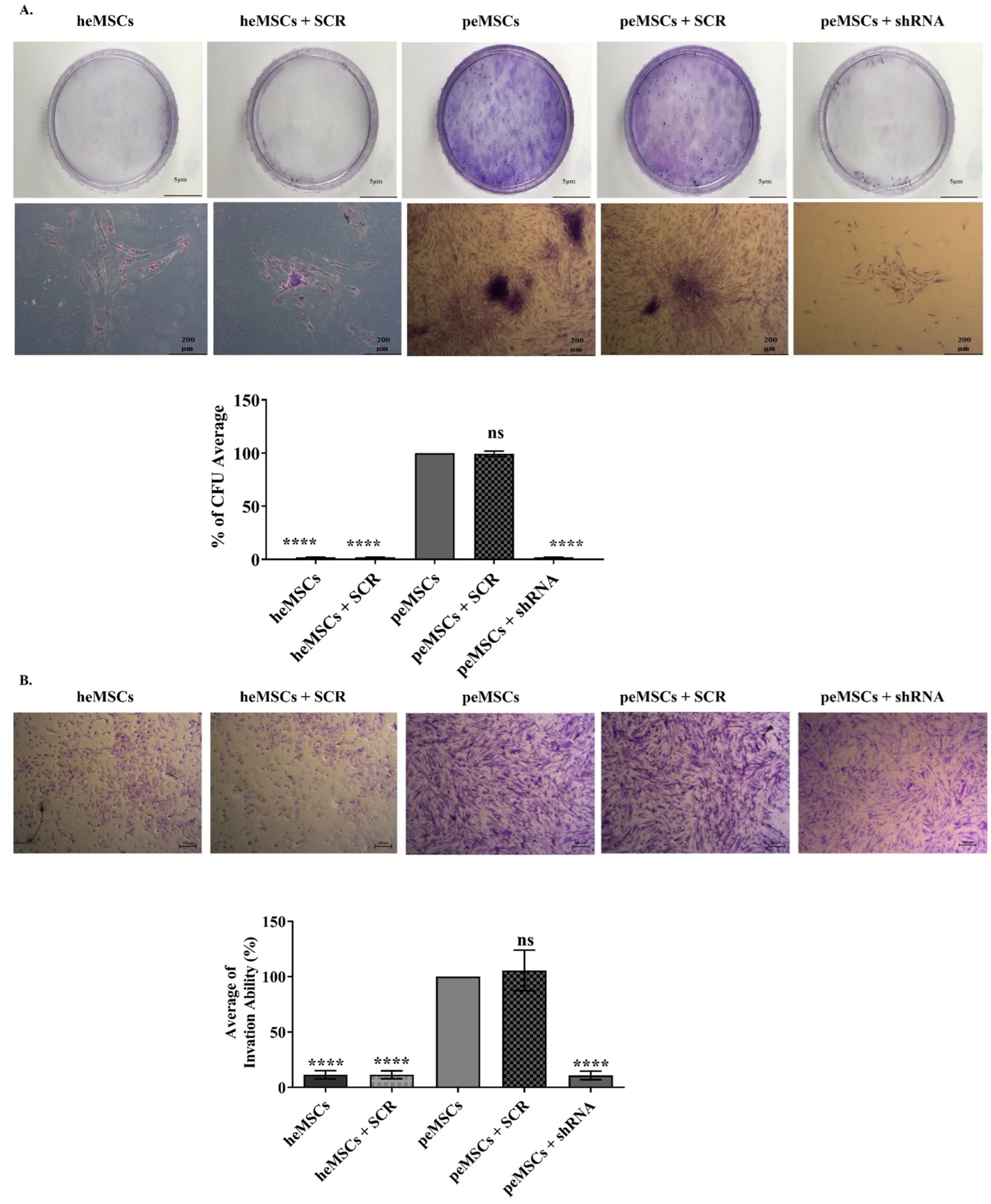
Analysis of clonogenic and invasive capacities following TG2 downregulation in eMSCs isolated from five healthy donors (heMSCs) and five patients with endometriosis (peMSCs). (**A**) Colony-forming unit (CFU) assay performed using untreated eMSCs and eMSCs transduced with either the scramble (SCR) control or TG2 shRNA. Representative images acquired on day 21, together with the corresponding quantitative analysis, are shown. (**B**) Transwell invasion assay performed under the corresponding experimental conditions. Representative images acquired after 18 h, together with the corresponding quantitative analysis, are shown. Data are presented as mean ± SD. The corresponding individual CFU and Transwell invasion images from the five independent donor-derived samples in each group (G1–G5) are provided in Supplementary Figure S10. Statistical significance is indicated as ns (not significant) and **** *p* ≤ 0.0001.

**Table 1:** Mean expression (%) of mesenchymal and hematopoietic stem cell surface markers in eMSCs isolated from five healthy donors (heMSCs) and five patients with endometriosis (peMSCs). Data are presented as mean ± SD. **** were used for *p* ≤ 0.0001 and ns was used for not significant.

	heMSCs	peMSCs	
	Average Expression % ± SD	Average Expression % ± SD	*p* Value
CD146	42.7 ± 3.1	75.5 ± 2.5	**** (*p* ≤ 0.0001)
W5C5	44.6 ± 2.4	74.0 ± 6.3	**** (*p* ≤ 0.0001)
PDGFR	43.4 ± 4.1	74.0 ± 2.3	**** (*p* ≤ 0.0001)
CD44	92.0 ± 4.3	94.1 ± 4.1	ns
CD29	93.9 ± 5.4	95.7 ± 1.6	ns
ITGβ-1	92.2 ± 7.6	97.9 ± 0.8	ns
CD73	91.9 ± 6.1	96.7 ± 2.0	ns
CD90	94.9 ± 2.67	97.7 ± 2.0	ns
CD105	90.9 ± 5.7	87.4 ± 3.7	ns
CD14	1.8 ± 0.9	1.4 ± 0.4	ns
CD31	1.1 ± 0.5	1.2 ± 0.2	ns
CD34	1.6 ± 0.8	1.6 ± 0.4	ns
CD45	1.4 ± 0.3	1.0 ± 0.4	ns

**Table 2:** Mean expression (%) of mesenchymal stem cell–associated and hematopoietic lineage surface markers in eMSCs following SCR or TG2 shRNA treatment. Data are presented as mean ± SD. Statistical comparisons were performed between heMSCs and heMSCs + SCR, and between peMSCs + SCR and peMSCs + shRNA. **** were used for *p* ≤ 0.0001; ns was used for not significant.

Marker	heMSCs	heMSCs + SCR	*p* Value	peMSCs + SCR	peMSCs + shRNA	*p* Value
CD146	46.2 ± 3.2	45.9 ± 3.1	ns	71.4 ± 5.1	35.5 ± 5.4	**** (*p* ≤ 0.0001)
W5C5	44.9 ± 4.1	45.2 ± 3.7	ns	76.6 ± 2.5	42.1 ± 3.4	**** (*p* ≤ 0.0001)
PDGFR	43.1 ± 3.0	42.9 ± 4.2	ns	78.5 ± 0.5	34.8 ± 4.0	**** (*p* ≤ 0.0001)
CD44	89.4 ± 5.2	90.5 ± 5.3	ns	89.1 ± 6.6	90.1 ± 7.6	ns
CD29	92.6 ± 4.5	91.3 ± 5.8	ns	92.5 ± 3.3	93.3 ± 5.6	ns
ITGβ-1	94.2 ± 2.8	94.1 ± 2.9	ns	93.6 ± 3.6	93.7 ± 3.4	ns
CD73	94.4 ± 4.8	94.7 ± 4.7	ns	90.8 ± 6.8	89.7 ± 7.4	ns
CD90	92.7 ± 1.8	91.2 ± 1.2	ns	88.2 ± 7.3	87.9 ± 8.2	ns
CD105	90.8 ± 6.4	91.6 ± 6.5	ns	93.0 ± 5.4	95.8 ± 4.5	ns
CD14	2.2 ± 1.1	1.9 ± 1.1	ns	1.7 ± 0.7	1.9 ± 0.9	ns
CD31	1.7 ± 0.9	1.6 ± 0.8	ns	1.7 ± 0.4	2.0 ± 0.3	ns
CD34	2.1 ± 0.8	2.1 ± 1.0	ns	2.0 ± 0.9	2.0 ± 0.9	ns
CD45	1.3 ± 0.5	0.9 ± 0.2	ns	1.7 ± 0.4	1.7 ± 0.3	ns

## Data Availability

The original contributions presented in this study are included in the article. Further inquiries can be directed at the corresponding author.

## Supplementary Materials

The following supporting information can be downloaded at: https://www.mdpi.com/article/10.3390/ph19101520/s1. Supplementary Figure S1. Flow cytometric immunophenotypic characterization of endometrial mesenchymal stem cells isolated from five healthy donors (heMSCs). Supplementary Figure S2. Flow cytometric immunophenotypic characterization of endometrial mesenchymal stem cells isolated from five patients with endometriosis (peMSCs). Supplementary Figure S3. Individual donor-/patient-specific TG2 mRNA expression in endometrial mesenchymal stem cells following TG2 shRNA-mediated knockdown. Supplementary Figure S4. Individual flow cytometric immunophenotypic characterization of endometrial mesenchymal stem cells isolated from five healthy donors (heMSCs) before and after scrambled shRNA (SCR) transduction. Supplementary Figure S5. Individual flow cytometric immunophenotypic characterization of endometrial mesenchymal stem cells isolated from five patients with endometriosis (peMSCs) before and after TG2 shRNA transduction. Supplementary Figure S6. Individual wound healing (scratch) assay images showing cell migration and wound closure at 0, 6, and 12 h in endometrial mesenchymal stem cells obtained from five independent healthy donors and five patients with endometriosis. Supplementary Figure S7. Individual gelatin zymography results demonstrating MMP-2 and MMP-9 gelatinolytic activities in endometrial mesenchymal stem cells obtained from five independent healthy donors and five patients with endometriosis. Supplementary Figure S8. Individual Western Blot analyses showing the effects of TG2 shRNA-mediated knockdown on the expression of cell cycle regulatory proteins in endometrial mesenchymal stem cells obtained from five independent healthy donors and five patients with endometriosis. Supplementary Figure S9. Individual cell cycle analyses performed using the Cytell Imaging System in endometrial mesenchymal stem cells obtained from five independent healthy donors and five patients with endometriosis. Supplementary Figure S10. Individual colony-forming unit (CFU) assays and Transwell invasion assays performed in endometrial mesenchymal stem cells obtained from five independent healthy donors and five patients with endometriosis following TG2 shRNA-mediated knockdown. Supplementary Table S1. Demographic and clinical characteristics of healthy donors and patients with endometriosis. Supplementary Table S2-Figure 1/Table 1. Statistical design and effect-size estimates for the quantitative flow-cytometric comparisons presented in Figure 1 and Table 1. Supplementary Table S3. Statistical design and effect-size estimates for the principal quantitative comparisons. Supplementary Table S4. Statistical design and effect-size estimates for the flow-cytometric comparisons presented in Figure 7 and Table 2.

## Abbreviations

The following abbreviations are used in this manuscript:

TG2	Tissue transglutaminase 2
eMSCs	Endometrial mesenchymal stem cells
heMSCs	Healthy endometrial mesenchymal stem cells
peMSCs	Patient-derived endometrial mesenchymal stem cells
shRNA	Short hairpin RNA
SCR	Scramble control
Co-IP	Co-immunoprecipitation
MMPs	Matrix metalloproteinases
ECM	Extracellular matrix
ITGβ-1	Integrin β-1
SDC-4	Syndecan-4
BTC	Biotin-X-cadaverine
