# Supplementary material for: Tissue Transglutaminase (TG2) as a Candidate Biomarker and Potential Therapeutic Target in Endometriosis: Functional Evidence from shRNA-Mediated Downregulation of TG2 in Endometrial Mesenchymal Stem Cells
Source: Pharmaceuticals (Basel). 2026 Sep 25;19(10):1520. doi: 10.3390/ph19101520 (PMC13708216; doi:10.3390/ph19101520)
Supplement: Supplementary file 1 [file pharmaceuticals-19-01520-s001.zip › Sup Fig Captions.pdf]

Supplementary Figure S1. Flow cytometric immunophenotypic characterization of endometrial mesenchymal stem cells isolated from five healthy donors (heMSCs). Cells were analyzed for the percentage of positive cells expressing mesenchymal stem cell–associated surface markers (CD146, W5C5, PDGFR, CD44, CD29, ITGβ-1, CD73, CD90, and CD105) together with the hematopoietic lineage markers CD14, CD31, CD34, and CD45. Purple histograms indicate specific antibody staining, whereas green histograms represent the corresponding isotype controls.

Supplementary Figure S2. Flow cytometric immunophenotypic characterization of endometrial mesenchymal stem cells isolated from five patients with endometriosis (peMSCs). Cells were analyzed for the percentage of positive cells expressing mesenchymal stem cell–associated surface markers (CD146, W5C5, PDGFR, CD44, CD29, ITGβ-1, CD73, CD90, and CD105) together with the hematopoietic lineage markers CD14, CD31, CD34, and CD45. Purple histograms indicate specific antibody staining, whereas green histograms represent the corresponding isotype controls

Supplementary Figure S3. Individual donor-/patient-specific TG2 mRNA expression in endometrial mesenchymal stem cells following TG2 shRNA-mediated knockdown. TG2 mRNA expression in endometrial mesenchymal stem cells obtained from five independent healthy donors (heMSCs) and five patients with endometriosis (peMSCs). Individual donor-/patient-specific results (G-1–G-5) are shown in the left panel for heMSCs, heMSCs + scrambled shRNA control (SCR), peMSCs + SCR, and peMSCs + TG2 shRNA groups. TG2 mRNA expression was determined by RT-qPCR and normalized to 18S rRNA. The corresponding group averages calculated from the individual donor-/patient-specific datasets are presented in the right panel as mean ± SD. Statistical significance for the group-level comparisons is indicated in the right-hand panels. ****$p \leq 0.0001$; ns, not significant. The individual datasets shown in the left panels constitute the biological donor-/patient-specific data used to calculate the group averages and statistical analyses presented in the corresponding right-hand panels.

Supplementary Figure S4. Individual flow cytometric immunophenotypic characterization of endometrial mesenchymal stem cells isolated from five healthy donors (heMSCs) before and after scrambled shRNA (SCR) transduction. Cells were analyzed for the percentage of positive cells expressing mesenchymal stem cell–associated surface markers (CD146, W5C5, PDGFR, CD44, CD29, ITGB1, CD73, CD90, and CD105), together with the hematopoietic lineage markers CD14, CD31, CD34, and CD45, to evaluate the effect of SCR transduction on the immunophenotypic characteristics of heMSCs. Histograms show paired analyses of each individual donor, with heMSC samples represented by the purple histograms and the corresponding heMSCs + SCR samples represented by the green histograms.

Supplementary Figure S5. Individual flow cytometric immunophenotypic characterization of endometrial mesenchymal stem cells isolated from five patients with endometriosis (peMSCs) before and after TG2 shRNA transduction. Cells were analyzed for the percentage of positive cells expressing mesenchymal stem cell–associated surface markers (CD146, W5C5, PDGFR, CD44, CD29, ITGB1, CD73, CD90, and CD105), together with the hematopoietic lineage markers CD14, CD31, CD34, and CD45, to evaluate the effect of TG2 silencing on the immunophenotypic characteristics of peMSCs. Histograms show paired analyses of each individual patient sample, with peMSCs + SCR samples represented by the purple histograms and the corresponding peMSCs + shRNA samples represented by the green histograms.

Supplementary Figure S6. Individual wound healing (scratch) assay images showing cell migration and wound closure at 0, 6, and 12 h in endometrial mesenchymal stem cells obtained from five independent healthy donors and five patients with endometriosis. Each panel represents an individual donor/patient and includes the corresponding experimental groups: heMSCs, heMSCs + SCR, peMSCs, peMSCs + SCR, and peMSCs + shRNA. Images illustrate the progression of wound closure over time following scratch induction and were used for quantitative migration analysis presented in the main manuscript. Images were acquired using a 20× objective, and 200 µm scale bars were included.

Supplementary Figure S7. Individual gelatin zymography results demonstrating MMP-2 and MMP-9 gelatinolytic activities in endometrial mesenchymal stem cells obtained from five independent healthy donors and five patients with endometriosis. (A) Gelatin zymography images from each individual healthy donor and patient sample. For each healthy and patient sample (G-1 to G-5), five experimental groups are presented in the following order: **heMSCs, heMSCs + SCR, peMSCs, peMSCs + SCR, and peMSCs + shRNA**. Gelatin degradation is visualized as white lytic bands, with the upper bands corresponding to MMP-9 activity and the lower bands corresponding to MMP-2 activity. **(B)** Individual densitometric quantification of **MMP-2 gelatinolytic activity** obtained from the gelatin zymography assay for each heMSCs, heMSCs + SCR, peMSCs, peMSCs + SCR, and peMSCs + shRNA. **(C)** Individual densitometric quantification of **MMP-9 gelatinolytic activity** obtained from the gelatin zymography assay for each heMSCs, heMSCs + SCR, peMSCs, peMSCs + SCR, and peMSCs + shRNA. The individual datasets presented here constitute the raw donor-/patient-specific data used to calculate the group averages and statistical analyses shown in the corresponding main figure of the manuscript.

Supplementary Figure S8. Individual Western Blot analyses showing the effects of TG2 shRNA-mediated knockdown on the expression of cell cycle regulatory proteins in endometrial mesenchymal stem cells obtained from five independent healthy donors and five patients with endometriosis. (A–E) Individual Western blot images corresponding to donor/patient samples **G-1 to G-5**, respectively. For each donor/patient, protein expression was

evaluated in the following experimental groups: **heMSCs, heMSCs + SCR, peMSCs, peMSCs + SCR, and peMSCs + shRNA**. Protein levels of **Cyclin E1, CDK-2, Cyclin D3, CDK-4, and p27** were analyzed to assess alterations in cell cycle regulation following TG2 silencing. **β-Actin was used as the loading control to verify equal protein loading across all lanes.** The individual Western blot datasets presented here constitute the donor-/patient-specific data used for densitometric quantification and statistical analyses shown in the corresponding main figure of the manuscript.

Supplementary Figure S9. Individual cell cycle analyses performed using the Cytell Imaging System in endometrial mesenchymal stem cells obtained from five independent healthy donors and five patients with endometriosis. (A–E) Individual donor/patient datasets corresponding to samples **G-1 to G-5**, respectively. For each donor/patient, cell cycle distribution was evaluated in the following experimental groups: heMSCs, heMSCs + SCR, peMSCs, peMSCs + SCR, and peMSCs + shRNA. The upper panels show the individual cell cycle histograms generated by the Cytell Imaging System, whereas the lower panels present the corresponding percentages of cells distributed among the G0/G1, S, and G2/M phases of the cell cycle. The individual datasets presented here constitute the donor-/patient-specific data used to calculate the group averages and statistical analyses shown in the corresponding main figure of the manuscript.

Supplementary Figure S10. Individual colony-forming unit (CFU) assays and Transwell invasion assays performed in endometrial mesenchymal stem cells obtained from five independent healthy donors and five patients with endometriosis following TG2 shRNA-mediated knockdown. (A) Individual CFU assay images showing the colony-forming capacity of donor-/patient-derived cells in the following experimental groups: heMSCs, heMSCs + SCR, peMSCs, peMSCs + SCR, and peMSCs + shRNA. CFU images were acquired at the end of day 21 using a 1× stereo microscope; scale bars = 5 μm. (B) Individual Transwell invasion assay images demonstrating the invasive potential of the corresponding donor-/patient-derived cells following the indicated treatments. Transwell images were captured 18 h after seeding using a 20× objective; scale bars = 100 μm. The individual datasets presented here constitute the donor-/patient-specific data used to calculate the group averages and statistical analyses shown in the corresponding main figures of the manuscript.
