# Supplementary material for: Tissue Transglutaminase (TG2) as a Candidate Biomarker and Potential Therapeutic Target in Endometriosis: Functional Evidence from shRNA-Mediated Downregulation of TG2 in Endometrial Mesenchymal Stem Cells
Source: Pharmaceuticals (Basel). 2026 Sep 25;19(10):1520. doi: 10.3390/ph19101520 (PMC13708216; doi:10.3390/ph19101520)
Supplement: Supplementary file 1 [file pharmaceuticals-19-01520-s001.zip › Supp Tables for Statistical Detail Final.pdf]

Supplementary Table S1. Demographic and clinical characteristics of healthy donors and patients with endometriosis.

Characteristic	Healthy donors (n = 5)	Endometriosis patients (n = 5)
Age (years), mean ± SD	30.6 ± 4.5	32.0 ± 3.5
Parity	2 (1–3)	1 (0–1)
Dysmenorrhea, n (%)	2 (40%)	5 (100%)
Endometriosis stage	N/A	rASRM stage II-III (mild–moderate)

Supplementary Table S2 - Figure 1 / Table 1. Statistical design and effect-size estimates for the quantitative flow-cytometric comparisons presented in Figure 1 and Table 1.

Outcome	Biological replicates	Design	Statistical analysis	Mean difference (95% CI)	Hedges' g (95% CI)
**CD146: heMSCs vs. peMSCs**	n = 5 vs. n = 5	Independent/unpaired donor groups	Two-way ANOVA followed by Šidák's multiple-comparisons test, $p < 0.0001$	**−32.78 (−39.20 to −26.36)**	**−9.46 (−11.22 to −7.67)**
**W5C5: heMSCs vs. peMSCs**	n = 5 vs. n = 5	Independent/unpaired donor groups	Two-way ANOVA followed by Šidák's multiple-comparisons test, $p < 0.0001$	**−29.39 (−35.81 to −22.97)**	**−8.48 (−10.15 to −6.78)**

**PDGFR: heMSCs vs. peMSCs**	n = 5 vs. n = 5	Independent/unpaired donor groups	Two-way ANOVA followed by Šidák's multiple-comparisons test, $p < 0.0001$	**−30.61 (−37.03 to −24.19)**	**−8.83 (−10.53 to −7.10)**
**CD44: heMSCs vs. peMSCs**	n = 5 vs. n = 5	Independent/unpaired donor groups	Two-way ANOVA followed by Šidák's multiple-comparisons test, ns	**−2.050 (−8.469 to 4.369)**	**−0.59 (−1.82 to 0.64)**
**CD29: heMSCs vs. peMSCs**	n = 5 vs. n = 5	Independent/unpaired donor groups	Two-way ANOVA followed by Šidák's multiple-comparisons test, ns	**−1.720 (−8.139 to 4.699)**	**−0.50 (−1.73 to 0.74)**
**ITGβ1: heMSCs vs. peMSCs**	n = 5 vs. n = 5	Independent/unpaired donor groups	Two-way ANOVA followed by Šidák's multiple-comparisons test, ns	**−5.730 (−12.15 to 0.6895)**	**−1.65 (−2.90 to −0.40)**
**CD73: heMSCs vs. peMSCs**	n = 5 vs. n = 5	Independent/unpaired donor groups	Two-way ANOVA followed by Šidák's multiple-comparisons test, ns	**−4.840 (−11.26 to 1.579)**	**−1.40 (−2.64 to −0.15)**
**CD90: heMSCs vs. peMSCs**	n = 5 vs. n = 5	Independent/unpaired donor groups	Two-way ANOVA followed by Šidák's multiple-comparisons test, ns	**−2.840 (−9.259 to 3.579)**	**−0.82 (−2.05 to 0.42)**

**CD105: heMSCs vs. peMSCs**	n = 5 vs. n = 5	Independent/unpaired donor groups	Two-way ANOVA followed by Šidák's multiple-comparisons test, ns	**3.480 (−2.939 to 9.899)**	**1.00 (−0.24 to 2.24)**
**CD14: heMSCs vs. peMSCs**	n = 5 vs. n = 5	Independent/unpaired donor groups	Two-way ANOVA followed by Šidák's multiple-comparisons test, ns	**0.360 (−6.059 to 6.779)**	**0.10 (−1.13 to 1.33)**
**CD31: heMSCs vs. peMSCs**	n = 5 vs. n = 5	Independent/unpaired donor groups	Two-way ANOVA followed by Šidák's multiple-comparisons test, ns	**−0.070 (−6.489 to 6.349)**	**−0.02 (−1.25 to 1.21)**
**CD34: heMSCs vs. peMSCs**	n = 5 vs. n = 5	Independent/unpaired donor groups	Two-way ANOVA followed by Šidák's multiple-comparisons test, ns	**0.000 (−6.419 to 6.419)**	**0.00 (−1.23 to 1.23)**
**CD45: heMSCs vs. peMSCs**	n = 5 vs. n = 5	Independent/unpaired donor groups	Two-way ANOVA followed by Šidák's multiple-comparisons test, ns	**0.450 (−5.969 to 6.869)**	**0.13 (−1.10 to 1.36)**

For Figure 2, the distinction between technical and biological replication has been clarified. In assays performed using replicate wells, such as the RT-qPCR analysis in Figure 2A and the BTC transamidase activity assay in Figure 2C, measurements for each donor-derived culture were performed in three technical replicate wells. Where p values are shown in the individual donor panels, these values reflect assay-level comparisons based on technical replicates obtained from the same donor-derived culture and were not treated as independent biological observations. The technical replicates were averaged within each donor to generate a single donor-level value, and group-level statistical analyses were subsequently performed using **five independent heMSC cultures and five independent peMSC cultures (n = 5 biological replicates per group).**

In contrast, for the Western blot analysis presented in Figure 2B, TG2 protein expression was evaluated independently in each heMSC- and peMSC-derived culture, yielding one normalized densitometric value for each donor. Accordingly, separate p values were not assigned to the individual

G1–G5 displays, because each donor represents a single independent biological observation and an individual donor-level value cannot by itself support statistical inference. The group-level comparison of TG2 protein expression was instead performed using the values obtained from the five independent heMSC and five independent peMSC donor-derived cultures.

The same analytical principle was applied to other assays performed using replicate wells, including RT-qPCR, BTC incorporation, WST-1, and similar quantitative assays. Technical replicates were first averaged within each donor, and the resulting donor-level means were then used as the independent biological observations for group-level statistical analysis. Thus, technical replicates were not used to increase the biological sample size.

The statistical analysis used for the principal group-level comparisons, together with the mean difference and its 95% confidence interval [mean difference (95% CI)] and Hedges' g with its corresponding 95% confidence interval [Hedges' g (95% CI)], has now been provided in **Supplementary Table S3**. These additional estimates are based on the five independent donor-level biological replicates per group and are presented as complementary measures of effect magnitude and precision.

Supplementary Table S3. Statistical design and effect-size estimates for the principal quantitative comparisons

**Outcome**	**Biological replicates**	**Design**	**Statistical analysis**	**Mean difference (95% CI)**	**Hedges' g (95% CI)**
**Figure 2 – Average of TG2 mRNA**	n = 5 heMSC vs. n = 5 peMSC	Independent/unpaired donor groups	Unpaired Student's *t*-test (one-tailed), $p = 0.0009$	−6.391 (−9.628 to −3.155)	−2.60 (−4.25 to −0.89)

**Figure 2 – Average of TG2 protein expression**	n = 5 heMSC vs. n = 5 peMSC	Independent/unpaired donor groups	Welch's unpaired *t*-test (two-tailed), $p < 0.0001$	−3.671 (−4.037 to −3.306)	−14.98 (−22.26 to −7.72)
**Figure 2 – Average of TG2 transamidase activity**	n = 5 heMSC vs. n = 5 peMSC	Independent/unpaired donor groups	Unpaired Student's *t*-test (two-tailed), $p < 0.0001$	1.027 (0.9115 to 1.142)	11.72 (5.99 to 17.45)
**Figure 3A – Average of MMP-2 mRNA**	n = 5 heMSC vs. n = 5 peMSC	Independent/unpaired donor groups	Unpaired Student's *t*-test (two-tailed), $p < 0.0001$	−1.367 (−1.590 to −1.145)	−8.09 (−12.13 to −4.04)
**Figure 3A – Average of MMP-9 mRNA**	n = 5 heMSC vs. n = 5 peMSC	Independent/unpaired donor groups	Unpaired Student's *t*-test (two-tailed), $p < 0.0001$	−1.681 (−1.800 to −1.562)	−18.61 (−27.62 to −9.63)
**Figure 3B – Average of MMP-2 activity (zymography)**	n = 5 heMSC vs. n = 5 peMSC	Independent/unpaired donor groups	Two-way ANOVA followed by Šidák's multiple-comparisons test, $p < 0.0001$	−0.730 (−0.847 to −0.613)	−9.27 (−12.64 to −5.86)
**Figure 3B – Average of MMP-9 activity (zymography)**	n = 5 heMSC vs. n = 5 peMSC	Independent/unpaired donor groups	Two-way ANOVA followed by Šidák's multiple-comparisons test, $p < 0.0001$	−0.390 (−0.507 to −0.273)	−4.95 (−7.00 to −2.86)

**Figure 4A – Average of TG2/ITGβ1 Co-IP**	n = 5 heMSC vs. n = 5 peMSC	Independent/unpaired donor groups	Unpaired Student's *t*-test (two-tailed), $p < 0.0001$	−5.593 (−7.380 to −3.806)	−4.12 (−6.37 to −1.82)
**Figure 4B – Average of TG2/SDC-4 Co-IP**	n = 5 heMSC vs. n = 5 peMSC	Independent/unpaired donor groups	Unpaired Student's *t*-test (two-tailed), $p < 0.0001$	−1.178 (−1.316 to −1.040)	−11.25 (−16.76 to −5.74)
**Figure 5A – Average of WST-1 cell number at 24 h**	n = 5 heMSC vs. n = 5 peMSC	Independent/unpaired donor groups	Two-way ANOVA followed by Šidák's multiple-comparisons test, $p = 0.0028$	−333.3 (−567.5 to −99.16)	−2.32 (−3.64 to −0.97)
**Figure 5A – Average of WST-1 cell number at 48 h**	n = 5 heMSC vs. n = 5 peMSC	Independent/unpaired donor groups	Two-way ANOVA followed by Šidák's multiple-comparisons test, $p < 0.0001$	−770.0 (−1004 to −535.8)	−5.36 (−7.12 to −3.56)
**Figure 5A – Average of WST-1 cell number at 72 h**	n = 5 heMSC vs. n = 5 peMSC	Independent/unpaired donor groups	Two-way ANOVA followed by Šidák's multiple-comparisons test, $p < 0.0001$	−1770 (−2004 to −1536)	−12.32 (−15.54 to −9.07)
**Figure 5A – Average of WST-1 cell number at 96 h**	n = 5 heMSC vs. n = 5 peMSC	Independent/unpaired donor groups	Two-way ANOVA followed by Šidák's multiple-	−1830 (−2064 to −1596)	−12.73 (−16.05 to −9.39)

			comparisons test, $p < 0.0001$		
**Figure 5B – Average of Cyclin E1 protein expression**	n = 5 heMSC vs. n = 5 peMSC	Independent/unpaired donor groups	Two-way ANOVA followed by Šidák's multiple-comparisons test, $p < 0.0001$	−2.410 (−3.015 to −1.805)	−6.66 (−8.54 to −4.74)
**Figure 5B – Average of CDK-2 protein expression**	n = 5 heMSC vs. n = 5 peMSC	Independent/unpaired donor groups	Two-way ANOVA followed by Šidák's multiple-comparisons test, $p < 0.0001$	−1.400 (−2.005 to −0.7947)	−3.87 (−5.33 to −2.37)
**Figure 5B – Average of Cyclin D3 protein expression**	n = 5 heMSC vs. n = 5 peMSC	Independent/unpaired donor groups	Two-way ANOVA followed by Šidák's multiple-comparisons test, $p < 0.0001$	−1.660 (−2.265 to −1.055)	−4.59 (−6.15 to −2.99)
**Figure 5B – Average of CDK-4 protein expression**	n = 5 heMSC vs. n = 5 peMSC	Independent/unpaired donor groups	Two-way ANOVA followed by Šidák's multiple-comparisons test, $p < 0.0001$	−1.530 (−2.135 to −0.9247)	−4.23 (−5.74 to −2.68)
**Figure 5B – Average of p27 protein expression**	n = 5 heMSC vs. n = 5 peMSC	Independent/unpaired donor groups	Two-way ANOVA followed by Šidák's multiple-comparisons test, $p \approx 0.0070$	0.770 (0.1647 to 1.375)	2.13 (0.81 to 3.42)

**Figure 6C – Average of TG2 protein level: heMSCs vs. heMSCs + SCR**	n = 5 vs. n = 5	Distinct experimental treatment conditions in donor-derived heMSC cultures	Ordinary one-way ANOVA followed by Šidák's multiple-comparisons test, $p = 0.9784$	0.1147 (−0.7272 to 0.9566)	0.22 (−0.97 to 1.40)
**Figure 6C – Average of TG2 protein level: heMSCs vs. peMSCs + SCR**	n = 5 vs. n = 5	Independent/unpaired donor groups	Ordinary one-way ANOVA followed by Šidák's multiple-comparisons test, $p < 0.0001$	−2.122 (−2.964 to −1.280)	−4.05 (−5.84 to −2.20)
**Figure 6C – Average of TG2 protein level: heMSCs vs. peMSCs + shRNA**	n = 5 vs. n = 5	Independent/unpaired donor groups	Ordinary one-way ANOVA followed by Šidák's multiple-comparisons test, $p = 0.9778$	0.1157 (−0.7262 to 0.9576)	0.22 (−0.97 to 1.40)
**Figure 6C – Average of TG2 protein level: peMSCs + SCR vs. peMSCs + shRNA**	n = 5 vs. n = 5	Distinct experimental treatment conditions in donor-derived peMSC cultures	Ordinary one-way ANOVA followed by Šidák's multiple-comparisons test, $p < 0.0001$	2.237 (1.351 to 3.123)	4.27 (2.36 to 6.12)
**Figure 8 – Average wound closure at 6 h: peMSCs vs. heMSCs**	n = 5 vs. n = 5	Independent/unpaired donor groups	Two-way ANOVA followed by Dunnett's multiple-comparisons test, $p < 0.0001$	28.71 (24.66 to 32.76)	11.19 (8.45 to 13.91)

**Figure 8 – Average wound closure at 6 h: peMSCs vs. heMSCs + SCR**	n = 5 vs. n = 5	Independent/unpaired donor groups	Two-way ANOVA followed by Dunnett's multiple-comparisons test, $p < 0.0001$	28.65 (24.60 to 32.70)	11.18 (8.43 to 13.89)
**Figure 8 – Average wound closure at 6 h: peMSCs vs. peMSCs + SCR**	n = 5 vs. n = 5	Distinct experimental treatment conditions in donor-derived peMSC cultures	Two-way ANOVA followed by Dunnett's multiple-comparisons test, ns	0.080 (−3.966 to 4.126)	0.03 (−1.19 to 1.25)
**Figure 8 – Average wound closure at 6 h: peMSCs vs. peMSCs + shRNA**	n = 5 vs. n = 5	Distinct experimental treatment conditions in donor-derived peMSC cultures	Two-way ANOVA followed by Dunnett's multiple-comparisons test, $p < 0.0001$	27.45 (23.40 to 31.50)	10.70 (8.05 to 13.32)
**Figure 8 – Average wound closure at 12 h: peMSCs vs. heMSCs**	n = 5 vs. n = 5	Independent/unpaired donor groups	Two-way ANOVA followed by Dunnett's multiple-comparisons test, $p < 0.0001$	93.70 (89.65 to 97.75)	36.54 (28.46 to 44.60)
**Figure 8 – Average wound closure at 12 h: peMSCs vs. heMSCs + SCR**	n = 5 vs. n = 5	Independent/unpaired donor groups	Two-way ANOVA followed by Dunnett's multiple-comparisons test, $p < 0.0001$	93.17 (89.12 to 97.22)	36.34 (28.30 to 44.35)

**Figure 8 – Average wound closure at 12 h: peMSCs vs. peMSCs + SCR**	n = 5 vs. n = 5	Distinct experimental treatment conditions in donor-derived peMSC cultures	Two-way ANOVA followed by Dunnett's multiple-comparisons test, ns	1.150 (−2.896 to 5.196)	0.45 (−0.77 to 1.67)
**Figure 8 – Average wound closure at 12 h: peMSCs vs. peMSCs + shRNA**	n = 5 vs. n = 5	Distinct experimental treatment conditions in donor-derived peMSC cultures	Two-way ANOVA followed by Dunnett's multiple-comparisons test, $p < 0.0001$	94.19 (90.14 to 98.24)	36.73 (28.61 to 44.83)
**Figure 9A – Average of MMP-2 mRNA: peMSCs vs. heMSCs**	n = 5 vs. n = 5	Independent/unpaired donor groups	Ordinary one-way ANOVA followed by Šidák's multiple-comparisons test, $p < 0.0001$	3.530 (2.743 to 4.317)	7.47 (4.86 to 10.04)
**Figure 9A – Average of MMP-2 mRNA: peMSCs vs. heMSCs + SCR**	n = 5 vs. n = 5	Independent/unpaired donor groups	Ordinary one-way ANOVA followed by Šidák's multiple-comparisons test, $p < 0.0001$	3.570 (2.783 to 4.357)	7.56 (4.92 to 10.15)
**Figure 9A – Average of MMP-2 mRNA: peMSCs vs. peMSCs + SCR**	n = 5 vs. n = 5	Distinct experimental treatment conditions in donor-derived peMSC cultures	Ordinary one-way ANOVA followed by Šidák's multiple-comparisons test, ns	0.130 (−0.6566 to 0.9166)	0.28 (−0.92 to 1.47)

**Figure 9A – Average of MMP-2 mRNA: peMSCs vs. peMSCs + shRNA**	n = 5 vs. n = 5	Distinct experimental treatment conditions in donor-derived peMSC cultures	Ordinary one-way ANOVA followed by Šidák's multiple-comparisons test, $p < 0.0001$	3.570 (2.783 to 4.357)	7.56 (4.92 to 10.15)
**Figure 9A – Average of MMP-9 mRNA: peMSCs vs. heMSCs**	n = 5 vs. n = 5	Independent/unpaired donor groups	Ordinary one-way ANOVA followed by Šidák's multiple-comparisons test, $p < 0.0001$	1.930 (1.451 to 2.409)	6.71 (4.31 to 9.07)
**Figure 9A – Average of MMP-9 mRNA: peMSCs vs. heMSCs + SCR**	n = 5 vs. n = 5	Independent/unpaired donor groups	Ordinary one-way ANOVA followed by Šidák's multiple-comparisons test, $p < 0.0001$	1.940 (1.461 to 2.419)	6.75 (4.33 to 9.12)
**Figure 9A – Average of MMP-9 mRNA: peMSCs vs. peMSCs + SCR**	n = 5 vs. n = 5	Distinct experimental treatment conditions in donor-derived peMSC cultures	Ordinary one-way ANOVA followed by Šidák's multiple-comparisons test, ns	−0.010 (−0.4886 to 0.4686)	−0.03 (−1.23 to 1.16)
**Figure 9A – Average of MMP-9 mRNA: peMSCs vs. peMSCs + shRNA**	n = 5 vs. n = 5	Distinct experimental treatment conditions in donor-derived peMSC cultures	Ordinary one-way ANOVA followed by Šidák's multiple-comparisons test, $p < 0.0001$	1.960 (1.481 to 2.439)	6.81 (4.38 to 9.20)

**Figure 9B – Average of MMP-2 activity: peMSCs vs. heMSCs**	n = 5 vs. n = 5	Independent/unpaired donor groups	Ordinary one-way ANOVA followed by Holm–Šidák's multiple-comparisons test, $p < 0.0001$	1.523 (1.338 to 1.708)	10.43 (6.99 to 13.84)
**Figure 9B – Average of MMP-2 activity: peMSCs vs. heMSCs + SCR**	n = 5 vs. n = 5	Independent/unpaired donor groups	Ordinary one-way ANOVA followed by Holm–Šidák's multiple-comparisons test, $p < 0.0001$	1.560 (1.375 to 1.745)	10.69 (7.18 to 14.17)
**Figure 9B – Average of MMP-2 activity: peMSCs vs. peMSCs + SCR**	n = 5 vs. n = 5	Distinct experimental treatment conditions in donor-derived peMSC cultures	Ordinary one-way ANOVA followed by Holm–Šidák's multiple-comparisons test, ns	0.0968 (−0.0884 to 0.2821)	0.66 (−0.55 to 1.87)
**Figure 9B – Average of MMP-2 activity: peMSCs vs. peMSCs + shRNA**	n = 5 vs. n = 5	Distinct experimental treatment conditions in donor-derived peMSC cultures	Ordinary one-way ANOVA followed by Holm–Šidák's multiple-comparisons test, $p < 0.0001$	1.658 (1.473 to 1.843)	11.35 (7.65 to 15.02)
**Figure 9B – Average of MMP-9 activity: peMSCs vs. heMSCs**	n = 5 vs. n = 5	Independent/unpaired donor groups	Ordinary one-way ANOVA followed by Šidák's multiple-comparisons test, $p < 0.0001$	0.7410 (0.5768 to 0.9051)	8.63 (5.70 to 11.52)

**Figure 9B – Average of MMP-9 activity: peMSCs vs. heMSCs + SCR**	n = 5 vs. n = 5	Independent/unpaired donor groups	Ordinary one-way ANOVA followed by Šidák's multiple-comparisons test, $p < 0.0001$	0.7320 (0.5679 to 0.8961)	8.53 (5.63 to 11.39)
**Figure 9B – Average of MMP-9 activity: peMSCs vs. peMSCs + SCR**	n = 5 vs. n = 5	Distinct experimental treatment conditions in donor-derived peMSC cultures	Ordinary one-way ANOVA followed by Šidák's multiple-comparisons test, ns	0.04375 (−0.1204 to 0.2079)	0.51 (−0.70 to 1.71)
**Figure 9B – Average of MMP-9 activity: peMSCs vs. peMSCs + shRNA**	n = 5 vs. n = 5	Distinct experimental treatment conditions in donor-derived peMSC cultures	Ordinary one-way ANOVA followed by Šidák's multiple-comparisons test, $p < 0.0001$	0.7720 (0.6079 to 0.9361)	9.00 (5.97 to 11.99)
**Figure 10A – Average of WST-1 cell number at 24 h: heMSCs vs. heMSCs + SCR**	n = 5 vs. n = 5	Distinct experimental treatment conditions in donor-derived heMSC cultures	Two-way ANOVA followed by Šidák's multiple-comparisons test, ns	25.18 (−571.7 to 622.0)	0.07 (−1.16 to 1.30)
**Figure 10A – Average of WST-1 cell number at 24 h: heMSCs vs. peMSCs**	n = 5 vs. n = 5	Independent/unpaired donor groups	Two-way ANOVA followed by Šidák's multiple-comparisons test, $p < 0.0001$	−2370 (−2966 to −1773)	−6.34 (−7.90 to −4.76)

**Figure 10A – Average of WST-1 cell number at 24 h: heMSCs vs. peMSCs + SCR**	n = 5 vs. n = 5	Independent/unpaired donor groups	Two-way ANOVA followed by Šidák's multiple-comparisons test, $p < 0.0001$	−2257 (−2853 to −1660)	−6.04 (−7.57 to −4.48)
**Figure 10A – Average of WST-1 cell number at 24 h: heMSCs vs. peMSCs + shRNA**	n = 5 vs. n = 5	Independent/unpaired donor groups	Two-way ANOVA followed by Šidák's multiple-comparisons test, ns	46.42 (−550.4 to 643.3)	0.12 (−1.10 to 1.35)
**Figure 10A – Average of WST-1 cell number at 48 h: heMSCs vs. heMSCs + SCR**	n = 5 vs. n = 5	Distinct experimental treatment conditions in donor-derived heMSC cultures	Two-way ANOVA followed by Šidák's multiple-comparisons test, ns	124.8 (−472.0 to 721.6)	0.33 (−0.90 to 1.56)
**Figure 10A – Average of WST-1 cell number at 48 h: heMSCs vs. peMSCs**	n = 5 vs. n = 5	Independent/unpaired donor groups	Two-way ANOVA followed by Šidák's multiple-comparisons test, $p < 0.0001$	−3000 (−3597 to −2403)	−8.03 (−9.76 to −6.27)
**Figure 10A – Average of WST-1 cell number at 48 h: heMSCs vs. peMSCs + SCR**	n = 5 vs. n = 5	Independent/unpaired donor groups	Two-way ANOVA followed by Šidák's multiple-comparisons test, $p < 0.0001$	−3018 (−3615 to −2421)	−8.07 (−9.81 to −6.31)

**Figure 10A – Average of WST-1 cell number at 48 h: heMSCs vs. peMSCs + shRNA**	n = 5 vs. n = 5	Independent/unpaired donor groups	Two-way ANOVA followed by Šidák's multiple-comparisons test, ns	374.6 (−222.2 to 971.4)	1.00 (−0.24 to 2.24)
**Figure 10A – Average of WST-1 cell number at 72 h: heMSCs vs. heMSCs + SCR**	n = 5 vs. n = 5	Distinct experimental treatment conditions in donor-derived heMSC cultures	Two-way ANOVA followed by Šidák's multiple-comparisons test, ns	85.40 (−511.4 to 682.2)	0.23 (−1.00 to 1.46)
**Figure 10A – Average of WST-1 cell number at 72 h: heMSCs vs. peMSCs**	n = 5 vs. n = 5	Independent/unpaired donor groups	Two-way ANOVA followed by Šidák's multiple-comparisons test, $p < 0.0001$	−5645 (−6241 to −5048)	−15.10 (−17.73 to −12.45)
**Figure 10A – Average of WST-1 cell number at 72 h: heMSCs vs. peMSCs + SCR**	n = 5 vs. n = 5	Independent/unpaired donor groups	Two-way ANOVA followed by Šidák's multiple-comparisons test, $p < 0.0001$	−5654 (−6251 to −5057)	−15.12 (−17.76 to −12.47)
**Figure 10A – Average of WST-1 cell number at 72 h: heMSCs vs. peMSCs + shRNA**	n = 5 vs. n = 5	Independent/unpaired donor groups	Two-way ANOVA followed by Šidák's multiple-comparisons test, ns	352.0 (−244.8 to 948.8)	0.94 (−0.30 to 2.18)

**Figure 10A – Average of WST-1 cell number at 96 h: heMSCs vs. heMSCs + SCR**	n = 5 vs. n = 5	Distinct experimental treatment conditions in donor-derived heMSC cultures	Two-way ANOVA followed by Šidák's multiple-comparisons test, ns	4.250 (−592.6 to 601.1)	0.01 (−1.22 to 1.24)
**Figure 10A – Average of WST-1 cell number at 96 h: heMSCs vs. peMSCs**	n = 5 vs. n = 5	Independent/unpaired donor groups	Two-way ANOVA followed by Šidák's multiple-comparisons test, $p < 0.0001$	−6375 (−6972 to −5778)	−17.05 (−19.95 to −14.13)
**Figure 10A – Average of WST-1 cell number at 96 h: heMSCs vs. peMSCs + SCR**	n = 5 vs. n = 5	Independent/unpaired donor groups	Two-way ANOVA followed by Šidák's multiple-comparisons test, $p < 0.0001$	−6206 (−6803 to −5609)	−16.60 (−19.44 to −13.74)
**Figure 10A – Average of WST-1 cell number at 96 h: heMSCs vs. peMSCs + shRNA**	n = 5 vs. n = 5	Independent/unpaired donor groups	Two-way ANOVA followed by Šidák's multiple-comparisons test, $p < 0.05$	699.3 (102.4 to 1296)	1.87 (0.60 to 3.13)
**Figure 10B – Average of Cyclin E1 protein expression: peMSCs vs. heMSCs**	n = 5 vs. n = 5	Independent/unpaired donor groups	Two-way ANOVA followed by Dunnett's multiple-comparisons test, $p < 0.0001$	1.008 (0.7474 to 1.270)	6.01 (4.51 to 7.48)
**Figure 10B – Average of Cyclin E1 protein**	n = 5 vs. n = 5	Independent/unpaired donor groups	Two-way ANOVA followed by Dunnett's multiple-	1.009 (0.7475 to 1.270)	6.02 (4.52 to 7.49)

**expression: peMSCs vs. heMSCs + SCR**			comparisons test, $p < 0.0001$		
**Figure 10B – Average of Cyclin E1 protein expression: peMSCs vs. peMSCs + SCR**	n = 5 vs. n = 5	Distinct experimental treatment conditions in donor-derived peMSC cultures	Two-way ANOVA followed by Dunnett's multiple-comparisons test, ns	−0.01203 (−0.2731 to 0.2491)	−0.07 (−1.30 to 1.16)
**Figure 10B – Average of Cyclin E1 protein expression: peMSCs vs. peMSCs + shRNA**	n = 5 vs. n = 5	Distinct experimental treatment conditions in donor-derived peMSC cultures	Two-way ANOVA followed by Dunnett's multiple-comparisons test, $p < 0.0001$	0.9861 (0.7250 to 1.247)	5.88 (4.40 to 7.35)
**Figure 10B – Average of CDK-2 protein expression: peMSCs vs. heMSCs**	n = 5 vs. n = 5	Independent/unpaired donor groups	Two-way ANOVA followed by Dunnett's multiple-comparisons test, $p < 0.0001$	0.9533 (0.6922 to 1.214)	5.69 (4.22 to 7.14)
**Figure 10B – Average of CDK-2 protein expression: peMSCs vs. heMSCs + SCR**	n = 5 vs. n = 5	Independent/unpaired donor groups	Two-way ANOVA followed by Dunnett's multiple-comparisons test, $p < 0.0001$	0.9411 (0.6800 to 1.202)	5.62 (4.15 to 7.06)
**Figure 10B – Average of CDK-2 protein expression: peMSCs vs. peMSCs + SCR**	n = 5 vs. n = 5	Distinct experimental treatment conditions in donor-derived peMSC cultures	Two-way ANOVA followed by Dunnett's multiple-comparisons test, ns	−0.01319 (−0.2743 to 0.2479)	−0.08 (−1.31 to 1.15)

**Figure 10B – Average of CDK-2 protein expression: peMSCs vs. peMSCs + shRNA**	n = 5 vs. n = 5	Distinct experimental treatment conditions in donor-derived peMSC cultures	Two-way ANOVA followed by Dunnett's multiple-comparisons test, $p < 0.0001$	0.9597 (0.6986 to 1.221)	5.72 (4.25 to 7.18)
**Figure 10B – Average of Cyclin D3 protein expression: peMSCs vs. heMSCs**	n = 5 vs. n = 5	Independent/unpaired donor groups	Two-way ANOVA followed by Dunnett's multiple-comparisons test, $p < 0.0001$	0.9687 (0.7076 to 1.230)	5.78 (4.30 to 7.23)
**Figure 10B – Average of Cyclin D3 protein expression: peMSCs vs. heMSCs + SCR**	n = 5 vs. n = 5	Independent/unpaired donor groups	Two-way ANOVA followed by Dunnett's multiple-comparisons test, $p < 0.0001$	0.9520 (0.6909 to 1.213)	5.68 (4.21 to 7.13)
**Figure 10B – Average of Cyclin D3 protein expression: peMSCs vs. peMSCs + SCR**	n = 5 vs. n = 5	Distinct experimental treatment conditions in donor-derived peMSC cultures	Two-way ANOVA followed by Dunnett's multiple-comparisons test, ns	−0.004103 (−0.2652 to 0.2570)	−0.02 (−1.25 to 1.21)
**Figure 10B – Average of Cyclin D3 protein expression: peMSCs vs. peMSCs + shRNA**	n = 5 vs. n = 5	Distinct experimental treatment conditions in donor-derived peMSC cultures	Two-way ANOVA followed by Dunnett's multiple-comparisons test, $p < 0.0001$	0.9732 (0.7121 to 1.234)	5.81 (4.33 to 7.27)

**Figure 10B – Average of CDK-4 protein expression: peMSCs vs. heMSCs**	n = 5 vs. n = 5	Independent/unpaired donor groups	Two-way ANOVA followed by Dunnett's multiple-comparisons test, $p < 0.0001$	0.9623 (0.7012 to 1.223)	5.74 (4.27 to 7.20)
**Figure 10B – Average of CDK-4 protein expression: peMSCs vs. heMSCs + SCR**	n = 5 vs. n = 5	Independent/unpaired donor groups	Two-way ANOVA followed by Dunnett's multiple-comparisons test, $p < 0.0001$	0.9623 (0.7012 to 1.223)	5.74 (4.27 to 7.20)
**Figure 10B – Average of CDK-4 protein expression: peMSCs vs. peMSCs + SCR**	n = 5 vs. n = 5	Distinct experimental treatment conditions in donor-derived peMSC cultures	Two-way ANOVA followed by Dunnett's multiple-comparisons test, ns	−0.02317 (−0.2843 to 0.2379)	−0.14 (−1.37 to 1.09)
**Figure 10B – Average of CDK-4 protein expression: peMSCs vs. peMSCs + shRNA**	n = 5 vs. n = 5	Distinct experimental treatment conditions in donor-derived peMSC cultures	Two-way ANOVA followed by Dunnett's multiple-comparisons test, $p < 0.0001$	0.9462 (0.6851 to 1.207)	5.65 (4.18 to 7.10)
**Figure 10B – Average of p27 protein expression: peMSCs vs. heMSCs**	n = 5 vs. n = 5	Independent/unpaired donor groups	Two-way ANOVA followed by Dunnett's multiple-comparisons test, $p < 0.0001$	−0.9738 (−1.235 to −0.7127)	−5.81 (−7.27 to −4.33)

**Figure 10B – Average of p27 protein expression: peMSCs vs. heMSCs + SCR**	n = 5 vs. n = 5	Independent/unpaired donor groups	Two-way ANOVA followed by Dunnett's multiple-comparisons test, $p < 0.0001$	−0.8112 (−1.072 to −0.5501)	−4.84 (−6.23 to −3.43)
**Figure 10B – Average of p27 protein expression: peMSCs vs. peMSCs + SCR**	n = 5 vs. n = 5	Distinct experimental treatment conditions in donor-derived peMSC cultures	Two-way ANOVA followed by Dunnett's multiple-comparisons test, ns	0.0002104 (−0.2609 to 0.2613)	0.00 (−1.23 to 1.23)
**Figure 10B – Average of p27 protein expression: peMSCs vs. peMSCs + shRNA**	n = 5 vs. n = 5	Distinct experimental treatment conditions in donor-derived peMSC cultures	Two-way ANOVA followed by Dunnett's multiple-comparisons test, $p < 0.0001$	−0.9580 (−1.219 to −0.6969)	−5.72 (−7.17 to −4.24)
**Figure 10C – Average of G0/G1 phase (%): peMSCs vs. heMSCs**	n = 5 vs. n = 5	Independent/unpaired donor groups	Two-way ANOVA followed by Dunnett's multiple-comparisons test, $p < 0.0001$	−43.68 (−48.37 to −38.99)	−14.60 (−17.49 to −11.72)
**Figure 10C – Average of G0/G1 phase (%): peMSCs vs. heMSCs + SCR**	n = 5 vs. n = 5	Independent/unpaired donor groups	Two-way ANOVA followed by Dunnett's multiple-comparisons test, $p < 0.0001$	−49.68 (−54.37 to −44.99)	−16.61 (−19.82 to −13.40)

**Figure 10C – Average of G0/G1 phase (%): peMSCs vs. peMSCs + SCR**	n = 5 vs. n = 5	Distinct experimental treatment conditions in donor-derived peMSC cultures	Two-way ANOVA followed by Dunnett's multiple-comparisons test, $p < 0.0001$	19.86 (15.17 to 24.55)	6.64 (4.93 to 8.35)
**Figure 10C – Average of G0/G1 phase (%): peMSCs vs. peMSCs + shRNA**	n = 5 vs. n = 5	Distinct experimental treatment conditions in donor-derived peMSC cultures	Two-way ANOVA followed by Dunnett's multiple-comparisons test, $p < 0.0001$	−39.02 (−43.71 to −34.33)	−13.05 (−15.68 to −10.41)
**Figure 10C – Average of S phase (%): peMSCs vs. heMSCs**	n = 5 vs. n = 5	Independent/unpaired donor groups	Two-way ANOVA followed by Dunnett's multiple-comparisons test, $p < 0.0001$	47.88 (43.19 to 52.57)	16.01 (12.89 to 19.12)
**Figure 10C – Average of S phase (%): peMSCs vs. heMSCs + SCR**	n = 5 vs. n = 5	Independent/unpaired donor groups	Two-way ANOVA followed by Dunnett's multiple-comparisons test, $p < 0.0001$	45.74 (41.05 to 50.43)	15.29 (12.29 to 18.29)
**Figure 10C – Average of S phase (%): peMSCs vs. peMSCs + SCR**	n = 5 vs. n = 5	Distinct experimental treatment conditions in donor-derived peMSC cultures	Two-way ANOVA followed by Dunnett's multiple-comparisons test, ns	−3.040 (−7.725 to 1.645)	−1.02 (−2.25 to 0.22)

**Figure 10C – Average of S phase (%): peMSCs vs. peMSCs + shRNA**	n = 5 vs. n = 5	Distinct experimental treatment conditions in donor-derived peMSC cultures	Two-way ANOVA followed by Dunnett's multiple-comparisons test, $p < 0.0001$	37.60 (32.91 to 42.29)	12.57 (10.01 to 15.13)
**Figure 10C – Average of G2/M phase (%): peMSCs vs. heMSCs**	n = 5 vs. n = 5	Independent/unpaired donor groups	Two-way ANOVA followed by Dunnett's multiple-comparisons test, ns	−2.760 (−7.445 to 1.925)	−0.92 (−2.16 to 0.31)
**Figure 10C – Average of G2/M phase (%): peMSCs vs. heMSCs + SCR**	n = 5 vs. n = 5	Independent/unpaired donor groups	Two-way ANOVA followed by Dunnett's multiple-comparisons test, ns	3.390 (−1.295 to 8.075)	1.13 (−0.11 to 2.37)
**Figure 10C – Average of G2/M phase (%): peMSCs vs. peMSCs + SCR**	n = 5 vs. n = 5	Distinct experimental treatment conditions in donor-derived peMSC cultures	Two-way ANOVA followed by Dunnett's multiple-comparisons test, $p < 0.0001$	−8.670 (−13.36 to −3.985)	−2.90 (−4.23 to −1.57)
**Figure 10C – Average of G2/M phase (%): peMSCs vs. peMSCs + shRNA**	n = 5 vs. n = 5	Distinct experimental treatment conditions in donor-derived peMSC cultures	Two-way ANOVA followed by Dunnett's multiple-comparisons test, ns	1.470 (−3.215 to 6.155)	0.49 (−0.74 to 1.72)
**Figure 11A – Average of CFU: peMSCs vs. heMSCs**	n = 5 vs. n = 5	Independent/unpaired donor groups	Ordinary one-way ANOVA followed by Šidák's multiple-comparisons test, $p < 0.0001$	98.02 (96.06 to 99.97)	83.54 (57.82 to 109.22)

**Figure 11A – Average of CFU: peMSCs vs. heMSCs + SCR**	n = 5 vs. n = 5	Independent/unpaired donor groups	Ordinary one-way ANOVA followed by Šidák's multiple-comparisons test, $p < 0.0001$	98.00 (96.04 to 99.95)	83.54 (57.82 to 109.22)
**Figure 11A – Average of CFU: peMSCs vs. peMSCs + SCR**	n = 5 vs. n = 5	Distinct experimental treatment conditions in donor-derived peMSC cultures	Ordinary one-way ANOVA followed by Šidák's multiple-comparisons test, ns	0.6681 (−1.285 to 2.621)	0.57 (−0.64 to 1.77)
**Figure 11A – Average of CFU: peMSCs vs. peMSCs + shRNA**	n = 5 vs. n = 5	Distinct experimental treatment conditions in donor-derived peMSC cultures	Ordinary one-way ANOVA followed by Šidák's multiple-comparisons test, $p < 0.0001$	98.13 (96.18 to 100.1)	83.66 (57.90 to 109.38)
**Figure 11B – Average of invasion ability (Transwell): peMSCs vs. heMSCs**	n = 5 vs. n = 5	Independent/unpaired donor groups	Ordinary one-way ANOVA followed by Šidák's multiple-comparisons test, $p < 0.0001$	88.49 (73.44 to 103.5)	9.79 (6.53 to 13.01)
**Figure 11B – Average of invasion ability (Transwell): peMSCs vs. heMSCs + SCR**	n = 5 vs. n = 5	Independent/unpaired donor groups	Ordinary one-way ANOVA followed by Šidák's multiple-comparisons test, $p < 0.0001$	88.49 (73.45 to 103.5)	9.79 (6.53 to 13.01)

**Figure 11B – Average of invasion ability (Transwell): peMSCs vs. peMSCs + SCR**	n = 5 vs. n = 5	Distinct experimental treatment conditions in donor-derived peMSC cultures	Ordinary one-way ANOVA followed by Šidák's multiple-comparisons test, ns	−5.639 (−20.69 to 9.411)	−0.62 (−1.82 to 0.59)
**Figure 11B – Average of invasion ability (Transwell): peMSCs vs. peMSCs + shRNA**	n = 5 vs. n = 5	Distinct experimental treatment conditions in donor-derived peMSC cultures	Ordinary one-way ANOVA followed by Šidák's multiple-comparisons test, $p < 0.0001$	89.11 (74.06 to 104.2)	9.86 (6.58 to 13.10)
**Supplementary Figure S3 – Average of TG2 mRNA: heMSCs vs. heMSCs + SCR**	n = 5 vs. n = 5	Distinct experimental treatment conditions in donor-derived heMSC cultures	Ordinary one-way ANOVA followed by Šidák's multiple-comparisons test, ns	0.05697 (−0.4460 to 0.5600)	0.18 (−1.00 to 1.36)
**Supplementary Figure S3 – Average of TG2 mRNA: heMSCs vs. peMSCs + SCR**	n = 5 vs. n = 5	Independent/unpaired donor groups	Ordinary one-way ANOVA followed by Šidák's multiple-comparisons test, $p < 0.0001$	−3.491 (−3.994 to −2.988)	−11.14 (−15.12 to −7.12)
**Supplementary Figure S3 – Average of TG2 mRNA: heMSCs vs. peMSCs + shRNA**	n = 5 vs. n = 5	Independent/unpaired donor groups	Ordinary one-way ANOVA followed by Šidák's multiple-comparisons test, ns	−0.02223 (−0.5252 to 0.4808)	−0.07 (−1.25 to 1.11)

Supplementary Table S4. Statistical design and effect-size estimates for the flow-cytometric comparisons presented in Figure 7 and Table 2.

Outcome	Biological replicates	Design	Statistical analysis	Mean difference (95% CI)	Hedges' g (95% CI)
**Figure 7A / Table 2 – CD146: heMSCs vs. heMSCs + SCR**	n = 5 vs. n = 5	Distinct experimental treatment conditions in donor-derived heMSC cultures	Two-way ANOVA followed by Šidák's multiple-comparisons test, ns	**0.260 (−6.478 to 6.998)**	**0.07 (−1.16 to 1.30)**
**Figure 7A / Table 2 – W5C5: heMSCs vs. heMSCs + SCR**	n = 5 vs. n = 5	Distinct experimental treatment conditions in donor-derived heMSC cultures	Two-way ANOVA followed by Šidák's multiple-comparisons test, ns	**−0.330 (−7.068 to 6.408)**	**−0.09 (−1.32 to 1.14)**
**Figure 7A / Table 2 – PDGFR: heMSCs vs. heMSCs + SCR**	n = 5 vs. n = 5	Distinct experimental treatment conditions in donor-derived heMSC cultures	Two-way ANOVA followed by Šidák's multiple-comparisons test, ns	**0.120 (−6.618 to 6.858)**	**0.03 (−1.20 to 1.26)**
**Figure 7A / Table 2 – CD44: heMSCs vs. heMSCs + SCR**	n = 5 vs. n = 5	Distinct experimental treatment conditions in donor-derived heMSC cultures	Two-way ANOVA followed by Šidák's multiple-comparisons test, ns	**−1.110 (−7.848 to 5.628)**	**−0.31 (−1.54 to 0.93)**
**Figure 7A / Table 2 – CD29: heMSCs vs. heMSCs + SCR**	n = 5 vs. n = 5	Distinct experimental treatment conditions in donor-derived heMSC cultures	Two-way ANOVA followed by Šidák's multiple-comparisons test, ns	**1.310 (−5.428 to 8.048)**	**0.36 (−0.87 to 1.59)**

**Figure 7A / Table 2 – ITGβ1: heMSCs vs. heMSCs + SCR**	n = 5 vs. n = 5	Distinct experimental treatment conditions in donor-derived heMSC cultures	Two-way ANOVA followed by Šidák's multiple-comparisons test, ns	**0.080 (−6.658 to 6.818)**	**0.02 (−1.21 to 1.25)**
**Figure 7A / Table 2 – CD73: heMSCs vs. heMSCs + SCR**	n = 5 vs. n = 5	Distinct experimental treatment conditions in donor-derived heMSC cultures	Two-way ANOVA followed by Šidák's multiple-comparisons test, ns	**−0.320 (−7.058 to 6.418)**	**−0.09 (−1.32 to 1.14)**
**Figure 7A / Table 2 – CD90: heMSCs vs. heMSCs + SCR**	n = 5 vs. n = 5	Distinct experimental treatment conditions in donor-derived heMSC cultures	Two-way ANOVA followed by Šidák's multiple-comparisons test, ns	**1.540 (−5.198 to 8.278)**	**0.42 (−0.81 to 1.65)**
**Figure 7A / Table 2 – CD105: heMSCs vs. heMSCs + SCR**	n = 5 vs. n = 5	Distinct experimental treatment conditions in donor-derived heMSC cultures	Two-way ANOVA followed by Šidák's multiple-comparisons test, ns	**−0.780 (−7.518 to 5.958)**	**−0.21 (−1.45 to 1.02)**
**Figure 7A / Table 2 – CD14: heMSCs vs. heMSCs + SCR**	n = 5 vs. n = 5	Distinct experimental treatment conditions in donor-derived heMSC cultures	Two-way ANOVA followed by Šidák's multiple-comparisons test, ns	**0.240 (−6.498 to 6.978)**	**0.07 (−1.17 to 1.30)**
**Figure 7A / Table 2 – CD31: heMSCs vs. heMSCs + SCR**	n = 5 vs. n = 5	Distinct experimental treatment conditions in donor-derived heMSC cultures	Two-way ANOVA followed by Šidák's multiple-comparisons test, ns	**0.090 (−6.648 to 6.828)**	**0.02 (−1.21 to 1.26)**
**Figure 7A / Table 2 – CD34: heMSCs vs. heMSCs + SCR**	n = 5 vs. n = 5	Distinct experimental treatment conditions in donor-derived heMSC cultures	Two-way ANOVA followed by Šidák's multiple-comparisons test, ns	**0.030 (−6.708 to 6.768)**	**0.01 (−1.22 to 1.24)**

**Figure 7A / Table 2 – CD45: heMSCs vs. heMSCs + SCR**	n = 5 vs. n = 5	Distinct experimental treatment conditions in donor-derived heMSC cultures	Two-way ANOVA followed by Šidák's multiple-comparisons test, ns	**0.440 (−6.298 to 7.178)**	**0.12 (−1.11 to 1.35)**
**Figure 7B / Table 2 – CD146: peMSCs + SCR vs. peMSCs + shRNA**	n = 5 vs. n = 5	Distinct experimental treatment conditions in donor-derived peMSC cultures	Two-way ANOVA followed by Šidák's multiple-comparisons test, $p < 0.0001$	**35.89 (27.48 to 44.30)**	**7.91 (6.26 to 9.55)**
**Figure 7B / Table 2 – W5C5: peMSCs + SCR vs. peMSCs + shRNA**	n = 5 vs. n = 5	Distinct experimental treatment conditions in donor-derived peMSC cultures	Two-way ANOVA followed by Šidák's multiple-comparisons test, $p < 0.0001$	**34.54 (26.13 to 42.95)**	**7.61 (6.00 to 9.22)**
**Figure 7B / Table 2 – PDGFR: peMSCs + SCR vs. peMSCs + shRNA**	n = 5 vs. n = 5	Distinct experimental treatment conditions in donor-derived peMSC cultures	Two-way ANOVA followed by Šidák's multiple-comparisons test, $p < 0.0001$	**43.74 (35.33 to 52.15)**	**9.64 (7.83 to 11.44)**
**Figure 7B / Table 2 – CD44: peMSCs + SCR vs. peMSCs + shRNA**	n = 5 vs. n = 5	Distinct experimental treatment conditions in donor-derived peMSC cultures	Two-way ANOVA followed by Šidák's multiple-comparisons test, ns	**−1.070 (−9.475 to 7.335)**	**−0.24 (−1.48 to 1.00)**
**Figure 7B / Table 2 – CD29: peMSCs + SCR vs. peMSCs + shRNA**	n = 5 vs. n = 5	Distinct experimental treatment conditions in donor-derived peMSC cultures	Two-way ANOVA followed by Šidák's multiple-comparisons test, ns	**−0.840 (−9.245 to 7.565)**	**−0.19 (−1.42 to 1.05)**
**Figure 7B / Table 2 – ITGβ1: peMSCs + SCR vs. peMSCs + shRNA**	n = 5 vs. n = 5	Distinct experimental treatment conditions in donor-derived peMSC cultures	Two-way ANOVA followed by Šidák's multiple-comparisons test, ns	**−0.110 (−8.515 to 8.295)**	**−0.02 (−1.26 to 1.22)**

**Figure 7B / Table 2 – CD73: peMSCs + SCR vs. peMSCs + shRNA**	n = 5 vs. n = 5	Distinct experimental treatment conditions in donor-derived peMSC cultures	Two-way ANOVA followed by Šidák's multiple-comparisons test, ns	**1.190 (−7.215 to 9.595)**	**0.26 (−0.98 to 1.50)**
**Figure 7B / Table 2 – CD90: peMSCs + SCR vs. peMSCs + shRNA**	n = 5 vs. n = 5	Distinct experimental treatment conditions in donor-derived peMSC cultures	Two-way ANOVA followed by Šidák's multiple-comparisons test, ns	**0.350 (−8.055 to 8.755)**	**0.08 (−1.16 to 1.32)**
**Figure 7B / Table 2 – CD105: peMSCs + SCR vs. peMSCs + shRNA**	n = 5 vs. n = 5	Distinct experimental treatment conditions in donor-derived peMSC cultures	Two-way ANOVA followed by Šidák's multiple-comparisons test, ns	**−2.800 (−11.21 to 5.605)**	**−0.62 (−1.86 to 0.63)**
**Figure 7B / Table 2 – CD14: peMSCs + SCR vs. peMSCs + shRNA**	n = 5 vs. n = 5	Distinct experimental treatment conditions in donor-derived peMSC cultures	Two-way ANOVA followed by Šidák's multiple-comparisons test, ns	**−0.180 (−8.585 to 8.225)**	**−0.04 (−1.28 to 1.20)**
**Figure 7B / Table 2 – CD31: peMSCs + SCR vs. peMSCs + shRNA**	n = 5 vs. n = 5	Distinct experimental treatment conditions in donor-derived peMSC cultures	Two-way ANOVA followed by Šidák's multiple-comparisons test, ns	**−0.340 (−8.745 to 8.065)**	**−0.07 (−1.31 to 1.16)**
**Figure 7B / Table 2 – CD34: peMSCs + SCR vs. peMSCs + shRNA**	n = 5 vs. n = 5	Distinct experimental treatment conditions in donor-derived peMSC cultures	Two-way ANOVA followed by Šidák's multiple-comparisons test, ns	**−0.060 (−8.465 to 8.345)**	**−0.01 (−1.25 to 1.23)**
**Figure 7B / Table 2 – CD45: peMSCs + SCR vs. peMSCs + shRNA**	n = 5 vs. n = 5	Distinct experimental treatment conditions in donor-derived peMSC cultures	Two-way ANOVA followed by Šidák's multiple-comparisons test, ns	**0.050 (−8.355 to 8.455)**	**0.01 (−1.23 to 1.25)**

Each donor-derived eMSC culture was considered one biological replicate, with five independent donor-derived cultures analyzed in each group (G1–G5; n = 5 per group). The heMSC and peMSC groups were derived from different individuals and therefore represented independent biological groups. Mean differences are reported in the comparison direction specified in each Outcome entry. For the flow-cytometric analyses, two-way ANOVA followed by Šidák's multiple-comparisons test was used. Hedges' *g* values were derived from the corresponding model-based contrasts and pooled residual variance with small-sample correction; the associated 95% confidence intervals are reported as measures of standardized effect-size precision. For ANOVA-based multiple comparisons, the 95% confidence intervals for mean differences correspond to the multiplicity-adjusted intervals generated by the respective post hoc test, whereas the 95% confidence intervals for Hedges' g are nominal (unadjusted) effect-size confidence intervals.
